# Frequency-dependent functional connectivity within resting-state networks: An atlas-based MEG beamformer solution

**DOI:** 10.1016/j.neuroimage.2011.11.005

**Published:** 2012-02-15

**Authors:** Arjan Hillebrand, Gareth R. Barnes, Johannes L. Bosboom, Henk W. Berendse, Cornelis J. Stam

**Affiliations:** a Department of Clinical Neurophysiology and Magnetoencephalography Center, VU University Medical Center, Amsterdam, The Netherlands; b Wellcome Trust Centre for Neuroimaging, University College London, London WC1N 3BG, UK; c Onze Lieve Vrouwe Gasthuis, Amsterdam, The Netherlands; d Department of Neurology, VU University Medical Center, Amsterdam, The Netherlands

**Keywords:** Beamforming, Magnetoencephalography, Source modelling, Functional connectivity, Graph analysis, Networks, Phase lag index, Resting-state

## Abstract

The brain consists of functional units with more-or-less specific information processing capabilities, yet cognitive functions require the co-ordinated activity of these spatially separated units. Magnetoencephalography (MEG) has the temporal resolution to capture these frequency-dependent interactions, although, due to volume conduction and field spread, spurious estimates may be obtained when functional connectivity is estimated on the basis of the extra-cranial recordings directly. Connectivity estimates on the basis of reconstructed sources may similarly be affected by biases introduced by the source reconstruction approach.

Here we propose an analysis framework to reliably determine functional connectivity that is based around two main ideas: (i) functional connectivity is computed for a set of atlas-based ROIs in anatomical space that covers almost the entire brain, aiding the interpretation of MEG functional connectivity/network studies, as well as the comparison with other modalities; (ii) volume conduction and similar bias effects are removed by using a functional connectivity estimator that is insensitive to these effects, namely the Phase Lag Index (PLI).

Our analysis approach was applied to eyes-closed resting-state MEG data for thirteen healthy participants. We first demonstrate that functional connectivity estimates based on phase coherence, even at the source-level, are biased due to the effects of volume conduction and field spread. In contrast, functional connectivity estimates based on PLI are not affected by these biases. We then looked at mean PLI, or weighted degree, over areas and subjects and found significant mean connectivity in three (alpha, beta, gamma) of the five (including theta and delta) classical frequency bands tested. These frequency-band dependent patterns of resting-state functional connectivity were distinctive; with the alpha and beta band connectivity confined to posterior and sensorimotor areas respectively, and with a generally more dispersed pattern for the gamma band. Generally, these patterns corresponded closely to patterns of relative source power, suggesting that the most active brain regions are also the ones that are most-densely connected.

Our results reveal for the first time, using an analysis framework that enables the reliable characterisation of resting-state dynamics in the human brain, how resting-state networks of functionally connected regions vary in a frequency-dependent manner across the cortex.

## Highlights

► Introducing a framework for reliable estimation of interactions in anatomical space. ► Choice of connectivity estimator (PLI) removes biases caused by volume conduction. ► Use of atlas-based ROIs aides interpretation and comparison with other modalities. ► Cortical profiles of resting-state power and connectivity are frequency-specific. ► Highly active brain regions are also the ones that are most-densely connected.

## Introduction

The brain consists of billions of interconnected neurons, forming an extremely complex system (Tononi and Edelman, 1998; Tononi et al., 1998) in which clusters of neurons are organised as functional units with more-or-less specific information processing capabilities (e.g. Born and Bradley, 2005; Grodzinsky, 2000). Yet, cognitive functions require the coordinated activity of these spatially separated units, where the oscillatory nature of neuronal activity, and phase relations between units, may provide a possible mechanism (Fries, 2005; Varela et al., 2001). Not only has it been shown that rhythmic activity plays an important role in perception and sensori-motor systems (Arieli et al., 1996; Forss and Silen, 2001; Hari and Salmelin, 1997; Houweling et al., 2010; Kenet et al., 2003; Linkenkaer-Hansen et al., 2004; Mima et al., 2000), as well as in higher cognitive functions (Engel et al., 2001; Ward, 2003), but it has also been shown that patterns of resting-state oscillatory activity in patients with neurological disorders differ from those in healthy subjects, and that these differences correlate with cognitive performance (Uhlhaas and Singer, 2006).

Magnetoencephalography (MEG), with its high temporal resolution, can be used to characterise the (resting-state) networks formed by interacting sources of oscillatory activity (Bassett et al., 2006; Hyvarinen et al., 2010; Langheim et al., 2006; Liu et al., 2010). However, in many MEG studies the required estimation of functional connectivity is performed at the sensor-level, which impedes comparison with the rapidly growing literature on resting-state functional connectivity using functional Magnetic Resonance Imaging (fMRI; van den Heuvel and Hulshoff Pol, 2010). Another problem is that multiple recording sites pick up signals from a single source due to both the nature of the induced magnetic flux (see e.g. Domínguez et al., 2007) and volume conduction, which can lead to erroneous estimates of functional connectivity when these estimates are based on sensor-level measurements. Moreover, the signals originating from spatially separated brain areas are mixed at the sensor level, which can result in over/underestimation of synchronisation, where the exact effect is dependent on a complex interplay of modulations in source- and noise-power, source interactions, as well as relative position and orientation of the sources (e.g. Grasman et al., 2004; Meinecke et al., 2005; Schoffelen and Gross, 2009).

These limitations have provoked research into four different directions: i) using estimates of the expected functional connectivity that is due to volume conduction/field spread without true interactions, and subtracting such estimates from the measured functional connectivity (Nunez et al., 1997), or using such estimates to derive a statistical threshold for the measured interactions (e.g. Brookes et al., 2011a); ii) the development of functional connectivity estimators that are insensitive to these confounds of field spread and volume conduction (Nolte et al., 2004; Stam et al., 2007); iii) development of techniques such as DCM (Dynamic Causal Modelling; Friston et al., 2011; Moran et al., 2009) that, given a set of hypotheses, test between different bio-physically motivated source- or network-models based on their model evidence; and iv) investigations into the utility of functional connectivity analysis at the source level. The simplest approach here is to create a source model to project the (unaveraged) sensor data into source space (Hoechstetter et al., 2004), although the creation of such a source montage requires the availability of averaged evoked data (see Grasman et al., 2004 though). The construction of a source montage can also be achieved by using a combination of Independent Component Analysis (ICA) and a multivariate autoregressive (MVAR) model (Gomez-Herrero et al., 2008; Haufe et al., 2010), or using Principle Component Analysis (PCA), ICA or MVAR in combination with an inverse estimator (Cheung et al., 2010; Mantini et al., 2011; Marzetti et al., 2008; Nolte et al., 2009). Alternatively, functional networks can be directly estimated at the source level (Dossevi et al., 2008), although this efficient approach is only applicable when the source matrix is obtained though a distributed linear inversion, and when the source coupling can be described as a scalar product between the source signals.

Various modifications of linear estimators have been used to reconstruct the time-series for a large number of locations (de Pasquale et al., 2010; Ghuman et al., 2011; Harle et al., 2004; Jerbi et al., 2007; Lin et al., 2004), for a set of cortical patches (e.g. David et al., 2002, 2003; Gruber et al., 2006; Palva et al., 2010a, 2010b; Supp et al., 2007), or for a limited set of a-priori defined Regions-of-Interest (ROIs; e.g. Astolfi et al., 2007; Babiloni et al., 2005; De Vico Fallani et al., 2007), where the time-series were subsequently used for the estimation of functional connectivity.

A drawback of the above approaches is that the spatially smooth estimates of neuronal activity that are obtained contain widespread correlations between reconstructed source elements, so that estimates of functional connectivity between such sources are likely to be, as with sensor-level analysis, erroneous (David et al., 2002; Hui et al., 2010) and/or difficult to interpret.

Here we propose to use a source reconstruction approach that results in sharper 3-dimensional images of neuronal activity, known as beamforming, which has recently been used to map functional connectivity across the entire brain (Brookes et al., 2011a; Guggisberg et al., 2008; Hinkley et al., 2010; Hipp et al., 2011; Kujala et al., 2006, 2008; Martino et al., 2011; Wibral et al., 2011), or to characterise interactions between a few ROIs (Siegel et al., 2008); see also Ding et al. (2007) and Ioannides et al. (2002) for related approaches. We use beamforming to estimate time-series for a set of atlas-based ROIs that cover the brain, where the use of a standard brain-atlas aids the interpretation of our results, gives a robust platform for group-level statistics, and enables a straightforward comparison with results obtained using other modalities. Functional connectivity between these ROIs is then estimated, and we first demonstrate that the effects of volume conduction and biases introduced by the beamformer can be removed by using the Phase Lag Index (PLI) for the estimation of functional connectivity. Applying this approach to resting-state MEG data in healthy controls reveals clearly distinct frequency-band dependent patterns of resting-state functional connectivity. Generally, these patterns corresponded closely to patterns of relative source power, suggesting that the most active brain regions are also the ones that are most-densely connected.

## Methods

### Participants and recording protocol

We used previously analysed MEG data from 13 healthy subjects, where they formed part of studies on Parkinson's disease for which approval was obtained from the medical ethics committee of the VU University Medical Center. In these studies oscillatory power, as well as functional connectivity and network characteristics at the sensor level, were estimated and compared between healthy controls and demented and non-demented patients with Parkinson's disease (Bosboom et al., 2006, 2009).

All subjects gave written informed consent prior to participating. MEG data were acquired in the morning, using a 151-channel whole head MEG system (CTF Systems Inc., Port Coquitlam, Canada), situated in a magnetically shielded room (Vacuum-schmelze GmbH, Hanau, Germany). The data were sampled at 312.5 Hz, with a recording pass-band of 0–125 Hz, and a third-order software gradient was applied (Vrba and Robinson, 2002). Each session started with an approximately 5 minutes eyes-closed (EC) resting-state recording, followed by an approximately 5 minutes eyes-open (EO) recording. We only analysed the data recorded during the eyes-closed resting-state. Due to technical problems, 1–3 channels were discarded from the analysis (3, 3, and 7 datasets contained 148, 149, and 150 channels, respectively). For the construction of the beamformer weights, the eyes-closed data were band-pass filtered from 0.5 to 48 Hz, and after visual inspection, trials containing artefacts were removed. A time-window of, on average, 264.2 seconds (range: 175–360 s.) was used for the computation of the data covariance matrix. Broadband data were used for the estimation of the beamformer weights as this avoids overestimation of covariance between channels (Barnes and Hillebrand, 2003).

For each subject, an anatomical MRI of the head was obtained at 1 T (Impact, Siemens, Erlangen, Germany), with an in-plane resolution of 1 mm and slice thickness of 1.5 mm. Vitamin E capsules were placed at anatomical landmarks, the pre-auricular points and the nasion, to guide co-registration with the MEG data. In the MEG setting, three head position indicator coils were placed at the same fiducial locations, and these coils were activated at the start of each MEG acquisition. Head position and orientation were computed on the basis of the magnetic fields produced by these coils. Using these two corresponding sets of fiducial markers, the MEG and MRI coordinate systems were matched. The co-registered MRI was subsequently segmented, and the outline of the scalp was used to compute a multi-sphere head model (Huang et al., 1999) for the calculation of the lead-fields.

### Analysis

**Fig. 1:**
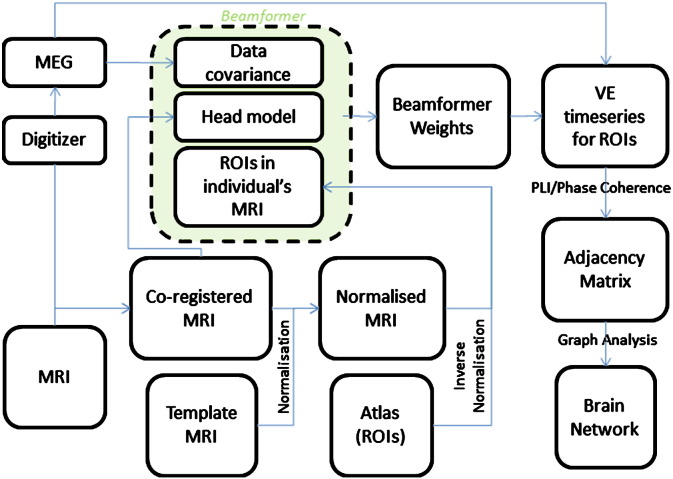
Flow chart of analysis steps. The anatomical MRI is co-registered with the MEG and subsequently spatially normalised to a template MRI. Voxels in the template MRI are labelled using the Talairach Daemon Database. Voxels with the same label are defined as a ROI and transformed to the individual's co-registered MRI. The volume conductor model, based on the co-registered MRI, together with the data covariance created from selected time-frequency windows in the MEG data, is used to compute beamformer weights for the target locations in these ROIs. The MEG data are then projected through the beamformer weights in order to create time-series (virtual electrodes) for these voxels. For each frequency band separately, a single time-series is constructed for each ROI (see Methods) and the functional connectivity between the different ROIs is estimated by computing the Phase Lag Index (PLI) or Phase Coherence. Graph theory can subsequently be applied to the resulting adjacency matrix in order to characterise the functional network formed by the interacting ROIs (see Supplementary material). Flow chart of analysis steps. The anatomical MRI is co-registered with the MEG and subsequently spatially normalised to a template MRI. Voxels in the template MRI are labelled using the Talairach Daemon Database. Voxels with the same label are defined as a ROI and transformed to the individual's co-registered MRI. The volume conductor model, based on the co-registered MRI, together with the data covariance created from selected time-frequency windows in the MEG data, is used to compute beamformer weights for the target locations in these ROIs. The MEG data are then projected through the beamformer weights in order to create time-series (virtual electrodes) for these voxels. For each frequency band separately, a single time-series is constructed for each ROI (see Methods) and the functional connectivity between the different ROIs is estimated by computing the Phase Lag Index (PLI) or Phase Coherence. Graph theory can subsequently be applied to the resulting adjacency matrix in order to characterise the functional network formed by the interacting ROIs (see Supplementary material).

Fig. 1 provides an overview of the analysis framework. The initial step is to project the MEG sensor signals in a meaningful way to a set of time-series of neuronal activation in the brain. To this end, we use a popular beamforming technique, known as Synthetic Aperture Magnetometry (SAM, Robinson and Vrba, 1999). The details of this technique are described elsewhere (Robinson and Vrba, 1999, see also Hillebrand et al. (2005) for a review), but we will describe its main features below.

### Beamformer analysis

The beamformer output at a target location, for a given orientation of the target source, can be defined as the weighted sum of the output of all (N) signal channels (van Veen et al., 1997), or mathematically:

(1) V=W·B,

with **V** the beamformer output (source strength in nAm), **W** the 1xN weight vector (units: nAm/T), and **B** the NxT_1_ matrix of the magnetic field (in Tesla) at the sensor locations at all (T_1_) latencies. **V** is often referred to as a virtual electrode, and has the same temporal resolution as the recorded MEG signals.

The weights determine the spatial filtering characteristics of the beamformer and are designed to increase the sensitivity to signals from a location of interest whilst reducing the contribution of signals from (noise) sources at different locations. The beamformer weights for a source at a location of interest are completely determined by the data covariance matrix and the forward solution (lead field) for the target source (see Mosher et al., 2003; Robinson and Vrba, 1999; van Drongelen et al., 1996; van Veen et al., 1997):

(2) $$V=C_{\text{j}}\;L^{\text{T}}\;C_{\text{b}}^{\text{-1}}\;Β\text{,}$$

with **C**_j_ the source current covariance matrix, **C**_b_ the data covariance matrix, **L** the lead field^1^ and T the matrix transpose.

Differences between various source reconstruction algorithms arise from the different assumptions that are made about the source current covariance matrix (see Hillebrand et al., 2005; Mosher et al., 2003). In the case of the beamforming approach it is assumed that all sources are linearly uncorrelated, i.e. **C**_j_ is a diagonal matrix, and that each diagonal element in **C**_j_, corresponding to a location θ, can be related to the measured data as follows (Mosher et al., 2003)

(3) $${\text{σ}}_{\text{θ}}^{2}={\left(L_{\text{θ}}^{\text{T}}\;C_{\text{b}}^{\text{-1}}\;L_{\text{θ}}\right)}^{-1}\text{.}$$

Eq. (3) is the crux of the beamformer algorithm. It is here that the source covariance **C**_j_ is estimated based on the data. Combination of the above three equations allows for the computation of the beamformer weights and beamformer output.

So far we assumed that the source orientation is known. SAM performs a search for the orientation that optimises the normalised beamformer output (Robinson and Vrba, 1999), defined as

(4) ƶθ2=PθNθ=WθTCbWθWθTΣWθ,

with **Σ** the sensor noise covariance matrix, N_θ_ the power of the projected sensor noise, and ƶθ the pseudo-Z statistic for location and orientation θ.

Alternatively, an eigendecomposition (Sekihara et al., 2004) can be used to determine the optimum source orientation, or one can simply compute the beamformer weights for the two tangential orientation components for each source (or all 3 orthogonal components in the case of electroencephalography (EEG); Sekihara et al., 2001; van Veen et al., 1997).

The standard approach is to subsequently form a 3-dimensional image of source activity (see Huang et al. (2004) for review) that quantifies the (change) in activity for a given time-segment in the MEG recording. Our approach however is to compute beamformer weights for those voxels that contained an anatomical label (see below), and use the complete time-series for all these virtual electrodes for further analysis, notably functional connectivity and network analysis.

### Defining ROIs for an individual

Our aim here is to define a set of regions-of-interest (ROIs) that is consistent across individuals. To this end, we spatially normalised a subject's anatomical MRI, using SPM99 (Friston et al., 1995), to a template MRI. The nonlinear transformation matrix that is necessary to perform this normalisation was stored.

The Talairach Daemon Database (TCDB), as built in the MEG/fMRI visualisation package mri3dX (https://cubric.psych.cf.ac.uk/Documentation/mri3dX), was used to label the voxels in the template MRI (Lancaster et al., 1997, 2000). We used the labels in the database at the level of description of Brodmann areas, and removed deeper structures, resulting in a set of 68 ROIs in template space (see Appendix A for a list of the labels that were used). The number of voxels per ROI varies, and is dependent on the chosen spatial sampling — voxels with side lengths of 5 mm were used in this study. The inverse of the nonlinear transformation matrix was applied to these ROIs, to create labelled target voxels in a subject's MRI for computation of the beamformer weights.

### Assigning time-series to a ROI

Once the beamformer weights were estimated, Eq. (1) was used to reconstruct the time-series for each voxel. These virtual electrode time-series exhibit a non-uniform projection of sensor noise (the weights increase with depth, but the sensor level noise remains constant throughout the volume). In order to compensate for this inherent bias, we therefore normalise each beamformer weight by its vector norm before reconstructing the time-series (Cheyne et al., 2007).

A ROI in a subject's MRI contains several voxels, all with their own time-series (virtual electrodes). The direction of each estimated virtual electrode is arbitrary (a source pointing inwards with negative amplitude produces the same external magnetic field as a source pointing outwards with positive amplitude), hence the estimated time-series for neighbouring virtual electrodes may have opposite polarities, rendering averaging of time-series across a ROI meaningless. We therefore proceeded to compute the spectrum for each virtual electrode time-series and divided the spectrum into the 5 classical EEG bands (delta (0.5–4 Hz), theta (4–8 Hz), alpha (8–13 Hz), beta (13–30 Hz), and gamma (30–48 Hz)). For each ROI and frequency band separately, we selected the voxel with maximum power in that frequency band, and used the time-series for this voxel for further analysis, resulting in a total of 5 sets of 68 time-series (one for each frequency band). Note that this procedure was carried out for each subject independently, such that the voxels that were selected to represent the ROIs were allowed to vary across subjects, mitigating the effects of co-registration, normalisation, and modelling errors (see e.g. Beal et al. (2010) for a similar strategy).

### Estimating functional connectivity between ROIs

Functional interactions between sources of oscillatory activity can be captured by quantifying the phase relationship between their time-series (see Pereda et al. (2005) for a review of coupling measures). Unfortunately, despite the assumptions underlying beamformers, the beamformer reconstructed sources may still show spurious, field spread and volume conduction related, interactions, which manifests itself as locking with zero-phase lags. To show that this is the case, and to demonstrate how this problem can be solved, we use both Phase Coherence (PC) and PLI to estimate functional connectivity between ROIs.

The Phase Coherence quantifies the phase coupling between two signals as follows (Mardia, 1972; Mormann et al., 2000):

(5) $$\text{PC}=\langle e^{\mathrm{i\Delta \varphi}}\rangle =\left|\frac{1}{\text{S}}\sum_{\text{k}=0}^{\text{S}-1}e^{i\Delta \varphi ({\text{t}}_{\text{k}})}\right|\text{,}$$

where Δ*Φ* is the phase difference between the instantaneous phases for the two time-series, defined in the interval [0, 2π], t_k_ are discrete time-steps and S is the number of samples.

Phase Coherence captures consistent phase differences and is, unlike coherence, not influenced by the amplitude of the signals. Phase Coherence is maximal when the phase difference has a constant value, whatever the value of this phase difference is, and is therefore equally sensitive to both trivial (zero-phase) and true (zero-phase and nonzero-phase) interactions.

In contrast, the PLI is defined as (Stam et al., 2007):

(6) $$\text{PLI}=\left|\langle \text{sign}\left[\sin\left(\Delta \varphi ({\text{t}}_{\text{k}})\right)\right]\rangle \right|\text{,}$$

where the phase difference is defined in the interval [− π, π] and <> denotes the mean value. The PLI is non-zero when there is an asymmetry in the distribution of the instantaneous phase differences, and therefore only quantifies non-trivial connections, at the expense of potentially discarding true interactions with zero-phase lag.

For the computation of the functional connectivity, using software developed by one of the authors (CS; Brainwave, version 0.8.92; http://home.kpn.nl/stam7883/brainwave.html), 5 artefact-free data-segments of 4096 samples were selected from the ROI time-series after careful visual inspection.

For each ROI we computed the mean PLI and Phase Coherence with all other areas. This is also known as the weighted degree or node strength in terms of graph theory (Rubinov and Sporns, 2010), where individual values reflect the importance of nodes in the network, the mean across ROIs indicates the total ‘wiring-cost’, and the distribution of degrees is an important marker of network development and resilience. We then computed the mean of this quantity across trials and subjects to get group mean node strength values per ROI.

### Statistics

In order to determine the significance of the empirical group mean values, we created 100 sets of null data by phase randomising (whilst maintaining the power spectra) the ROI time-series. For each realisation, this gave rise to 68 new ROI time-series per subject, which were analysed in exactly the same way as the recorded data, giving a mean PLI value per ROI. Taking the maximum mean-PLI value over ROIs on each realisation gave a null distribution of PLI corrected for multiple comparisons across the volume.

## Results

**Fig. 2:**
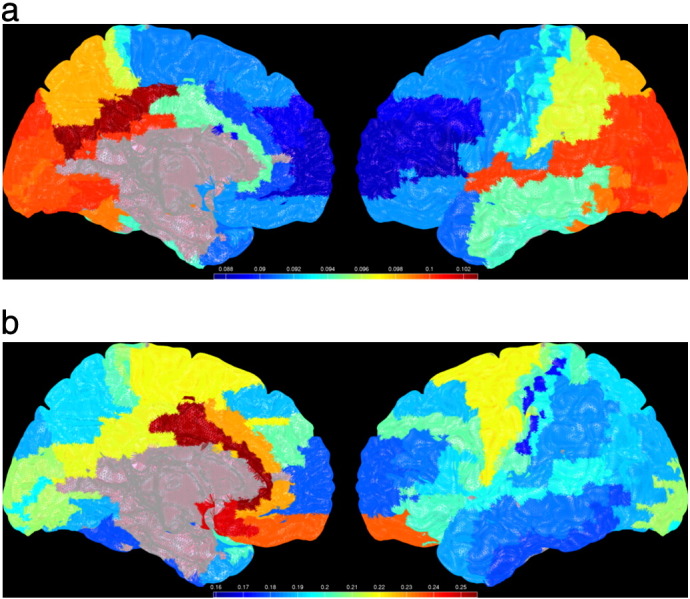
Mean PLI (upper panel) and mean Phase Coherence (lower panel) for the alpha band, displayed as a colour-coded map (unthresholded) on a schematic of the parcellated template brain.

Fig. 2 displays the mean functional connectivity for each ROI with all other ROIs. Note the differences between the maps for PLI and Phase Coherence, where Phase Coherence shows strongest functional connectivity for deeper structures, whereas the strongest PLI is found mainly for superficial areas in the occipital and parietal lobe and the superior temporal gyrus, as well as in the posterior cingulate.

**Fig. 3:**
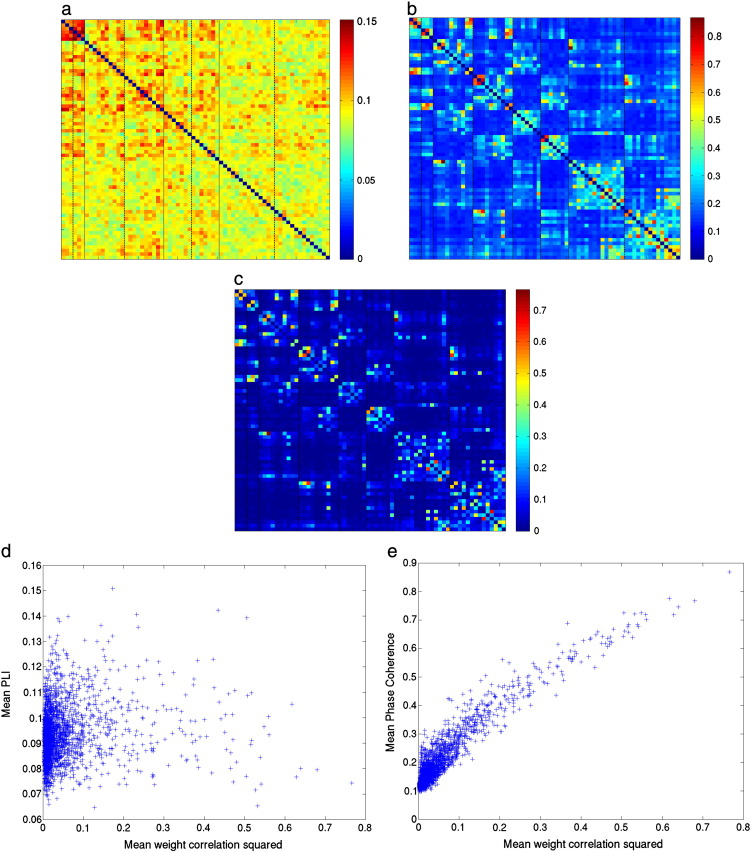
Functional connectivity and relationship with the beamformer weights for the alpha band. a) Mean PLI adjacency matrix. The separation between anatomical groupings (from left to right: occipital, parietal/central, temporal, frontal) is denoted by a solid line, the separation between left and right hemisphere within each anatomical grouping is denoted by a dotted line (see Appendix A for details); b) mean Phase Coherence adjacency matrix; c) mean (squared) correlation between beamformer weights for each ROI (with the diagonal set to zero). Each element in this matrix was computed as follows: for each subject, the square of the correlation between the beamformer weights for a ROI and another ROI was computed. The mean over subjects of this value was then computed; d) Scatter plot of the (squared) correlation between beamformer weights and the PLI and (e) Phase Coherence.

These differences can be explained by the different sensitivity of these measures to volume conduction/field spread (in the form of correlation between beamformer weights). Fig. 3 shows that there is a close correspondence between the weight correlations (Fig. 3c) and the Phase Coherence (Fig. 3b). This is confirmed in Fig. 3e, where a strong relationship between the weight correlations and Phase Coherence can be seen. This means that it is likely that any observed Phase Coherence could have been caused by the beamformer itself. Such a clear relationship between the mean PLI and the weight correlations is not observable in Fig. 3d.

**Fig. 4:**
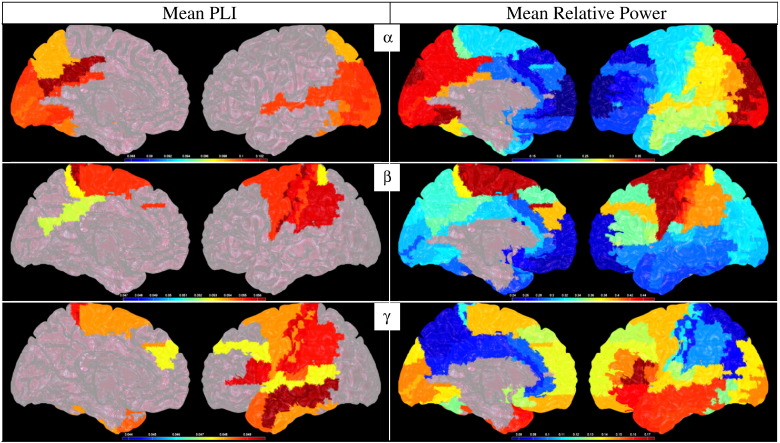
Mean PLI (left column, thresholded at p = 0.05) and mean relative power (right column) for alpha, beta and gamma bands (top to bottom), displayed as a colour-coded map on a schematic of the parcellated template brain (see Supplementary Fig. 4 for unthresholded results). See Appendix B for a list of the areas with significant mean PLI. (For interpretation of the references to colour in this figure legend, the reader is referred to the web version of this article.) Mean PLI (left column, thresholded at p = 0.05) and mean relative power (right column) for alpha, beta and gamma bands (top to bottom), displayed as a colour-coded map on a schematic of the parcellated template brain (see Supplementary Fig. 4 for unthresholded results). See Appendix B for a list of the areas with significant mean PLI.

Fig. 4 displays the mean PLI for the alpha, beta and gamma bands (the adjacency matrices themselves are given in Supplementary Fig. 1), as well as the mean relative power for these frequency bands. The mean PLI for the delta and theta bands did not reach statistical significance (see Supplementary Fig. 2 for the unthresholded PLI maps and maps of relative power for the delta and theta bands; Supplementary Fig. 3 shows results for the alpha1 and alpha2 bands).

The most strongly connected, as well as most strongly active, regions in the alpha band were the posterior cingulate, regions in visual cortex and parietal lobe, as well as in the superior and inferior temporal lobe.

The connectivity and power maps in the beta band showed a relatively restricted pattern of highly connected and strongly activated regions in the sensorimotor cortex, extending into the inferior parietal lobe and the dorsolateral prefrontal cortex (for source power).

In the gamma band, the most strongly connected regions were in the temporal lobe, sensorimotor cortex and the inferior frontal and parietal lobes. Dominant power was also found in these regions, but covered more of the frontal lobe and also included the visual cortex, with notably less power in the parietal lobe and posterior part of the sensorimotor cortex.

**Fig. 5:**
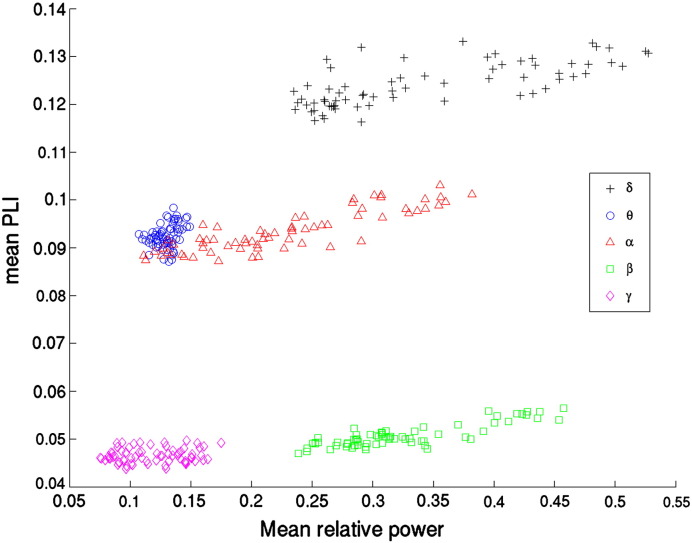
Mean PLI versus mean relative power for the different frequency bands. Note that there is a significant positive linear relationship between PLI and relative power, for all frequency bands, except the gamma band. Also note that, for each frequency band separately, the mean PLI varies over only a limited range, and that the variance in PLI that can be explained by source power is relatively small (R^2^ = 51%, 12%, 75%, 72% and 2% for the delta, theta, alpha, beta and gamma bands respectively).

It is clear from Fig. 4 that the patterns for PLI and power are similar, but that there are also notable exceptions where regions with high power do not have corresponding high PLI values, and vice versa (compare for example the power and PLI values for the occipital pole and parietal lobe in the gamma band). This is further illustrated in Fig. 5, where the relationship between PLI and power for the different frequency bands is shown. For all frequency bands, except the gamma band, there is a significant positive linear relationship between PLI and relative power (for delta, theta, alpha, beta and gamma bands respectively: F(1,66) = 69.98, *p* < 10^− 11^; F(1,66) = 8.99, *p* < 0.01; F(1,66) = 194.31, *p* < 10^− 15^; F(1,66) = 167.97, *p* < 10^− 15^; F(1,66) = 1.30, *p* = 0.26). However, the PLI values are not trivially related to the power values, as, for example, the relative power for the beta band and delta bands do not differ significantly (two-sample t(134) = − 1.56, *p* = 0.12), whereas the mean PLI values are significantly higher in the delta band than in the beta band (two-sample t(134) = 118.66, *p* < 10^− 15^). Similarly, the PLI for the theta and alpha bands do not differ significantly (two-sample t(134) = − 1.45, *p* = 0.15), whereas the mean power values are significantly higher in the alpha band than in the theta band (two-sample t(134) = 10.83, *p* < 10^− 15^).

## Discussion and conclusions

We have presented a robust method for assessing significant functional connectivity across a group of subjects that is insensitive to volume conduction and statistically well controlled. We first demonstrated that when using Phase Coherence, functional connectivity estimates, even at the source-level, are biased due to the effects of volume conduction and field spread. In contrast, functional connectivity estimates based on PLI are not affected by these biases. Subsequently, we used the method to show significantly higher than chance interactions between regions in three of the five frequency bands studied, revealing distinct frequency-band dependent patterns of functional connectivity across the brain.

### Alpha band

Strong connectivity was observed in the visual cortex and in the parietal and temporal lobes, consistent with the findings by Guggisberg et al. (2008). However, we could not find any direct evidence in the existing literature for the strong resting-state functional connectivity in the posterior cingulate for the alpha band.

Similarly, the dominant alpha power in the posterior part of the brain is consistent with the established patterns of eyes-closed resting-state alpha activity (e.g. Pfurtscheller, 1992; Rosanova et al., 2009). Additionally, several studies, using different methodologies and modalities, have reported activations similar to ours for the visual cortex, parietal and temporal lobe (Congedo et al., 2010; Srinivasan et al., 2006) as well as the posterior cingulate (Congedo et al., 2010).

### Beta band

We found strong resting-state beta-band functional connectivity in the sensorimotor cortex, in agreement with a previous MEG resting-state functional connectivity study (Brookes et al., 2011a). Moreover, the large body of literature on beta-band synchrony in sensorimotor systems involved in co-ordinated movement and posture (e.g. Farmer, 1998), suggests that these systems are also connected in this frequency band during rest. Further evidence for this hypothesis comes from single-pulse TMS studies that have shown that TMS synchronises the phase of beta-oscillations (van der Werf and Paus, 2006; van der Werf et al., 2006), and that stimulating one region of the sensorimotor system can induce activity in another part of this system (e.g. Caramia et al., 2000).

Our finding of strong resting-state beta power in sensori-motor cortex and inferior parietal lobe is consistent with studies that have revealed event-related beta-power reductions in these regions following somatosensory stimulation or movement (e.g. Gaetz and Cheyne, 2006; Jurkiewicz et al., 2006; Maratos et al., 2007; Taniguchi et al., 2000), or that showed that the beta-band is the natural frequency of these circuits (Rosanova et al., 2009). Our observed beta band power in the dorsolateral prefrontal cortex can tentatively be related to the reorienting of attention (Altamura et al., 2010).

### Gamma band

We had naively expected that gamma band PLI (like alpha band PLI) would predominate in the visual cortex. We found little literature to corroborate the resting-state functional connectivity in the temporal lobe, sensorimotor cortex and the inferior frontal and parietal lobe for the gamma band. Recent invasive electrode (Griffiths et al., 2010) and non-invasive studies on pitch perception (Sedley et al., 2012) have pointed to a functional role for the gamma band in the region of human auditory cortex. It would make sense that similar functional units are also to some degree engaged during the resting-state.

In terms of source power however, the observed dominant power in the frontal and temporal lobe is in agreement with previous EEG findings (Chen et al., 2008). Our observations are also in agreement with reports of both resting-state measures of gamma power in the primate (Leopold and Logothetis, 2003) and event-related changes in gamma power in visual cortex following visual stimulation (e.g. Adjamian et al., 2004; Hall et al., 2005b, 2005a; Hoogenboom et al., 2006).

#### Relationship between source power and functional connectivity

We found a positive relationship between the patterns of source power and functional connectivity, using a connectivity measure that is not sensitive to power or volume conduction. The fact that we found this relationship for all frequency bands (except the gamma band) suggests that there may be a general mechanism that explains this relationship. The simplest explanation is that a positive relationship between source power and connectivity was introduced by our analysis approach, since the functional connectivity measure that we used relies on accurate estimation of phase differences, and therefore on a high-enough SNR. Consequently, functional connectivity between regions with low source power may be missed or underestimated, introducing a bias towards a positive correlation between power and connectivity. However, we did find regions with strong connections despite low source power (e.g. parietal lobe for the gamma band, Fig. 4), hence it is unlikely that the positive relationship between source power and connectivity can be fully explained by such a methodological bias. One possible physiological mechanism that could provide an alternative explanation has been identified through modelling studies (Chawla et al., 1999, 2000), which have shown that increased mean spiking activity within two connected neuronal populations leads to increased intra- and interregional phase locking, or in other words, to simultaneous increases in power and functional connectivity. This coupling between functional connectivity and mean activity was mainly achieved through a reduction in mean membrane integration times as activity increased, which then introduced a bias towards synchronous firing (Chawla et al., 1999). Recent modelling work has also shown an amplitude dependency of interregional phase locking, albeit that this dependency was more complex (Daffertshofer and van Wijk, 2011).

### Methodological considerations

#### General analysis framework

A frequent criticism of sensor-level connectivity/network analysis is that the results are biased by the effects of volume conduction/field spread (Schoffelen and Gross, 2009). Our results show that this problem is not completely solved by going to source-space (Fig. 3), as it reveals itself, in our case, in the form of correlation between beamformer weights. As a solution, we used the PLI, which quantifies functional interactions that are not caused by volume conduction or common sources. The main reason for going to the source-level, in combination with an atlas-based analysis approach, is therefore that it provides a general framework that allows for a direction anatomical interpretation of MEG data, as well as a direct comparison with (functional) connectivity and network studies based on anatomical MRI (Diffusion Tensor Imaging, Voxel-based Morphometry) and functional MRI (e.g. Gong et al., 2009; van den Heuvel and Hulshoff Pol, 2010). We envisage that the understanding of resting-state networks will be much enhanced by such a combination of different modalities. Similarly, our approach enables the integration and direct comparison of data recorded with different MEG systems. Although we have focussed here on the eyes-closed resting-state, our approach can also be applied to compare patterns of oscillatory activity and functional networks for different cognitive states.

One can consider the choice of using a set of atlas-based ROIs as a compromise between all-to-all connectivity estimates (Schoffelen and Gross, 2011) and methods based on a-priori selection of a small number of regions (Astolfi et al., 2007; Friston et al., 2011; Siegel et al., 2008). The use of atlas-based ROIs does certainly compromise the potential spatial resolution, but could be the most efficient level of description given the inter-individual variability. Similarly, the use of predefined anatomical regions does mean that one samples from all sources rather than just those with highest source power (although within each ROI we did select the voxel with highest source power), thereby avoiding the danger that weakly activated, but strongly interacting, sources are missed (compare for example the connectivity and power in the parietal lobe for the gamma band (Fig. 4)).

#### Choice of source reconstruction approach

We chose to reconstruct the resting-state sources, and their time-series, using a beamforming approach, as this approach does not suffer from the problems associated with linear inverse solutions (widespread correlations between reconstructed source elements and problematic interpretation of reconstructed images of source power).

A potential limitation of the beamformer-based approach is that the beamformer weights are based on source power and interacting sources with small amplitude could therefore be missed (but see Fig. 4, gamma band power and connectivity). Moreover, the beamformer weights are designed so that the sensitivity to signals from a location of interest is increased, whilst reducing the contribution of signals from (noise) sources at different locations. As a consequence the contribution from sources that are perfectly linearly correlated is cancelled or underestimated (which might in particular be problematic for weak long-distance interactions). At first sight, this property of beamforming seems therefore at odds with our aim to estimate interactions between sources. However, there are several reasons why beamforming can still be applied in studies on functional connectivity: i) it is important to stress that only (zero-lag) linearly correlated sources are problematic for the beamformer. Non-linear measures of functional connectivity should therefore be less affected; ii) even for linearly correlated sources, there is a remarkable tolerance to deviations from the uncorrelatedness-assumption, such that source interdependencies can be accurately reconstructed even when these sources are correlated for 30–40% of the analysis time-window (Hadjipapas et al., 2005); iii) anatomical and electrophysiological data suggest that the uncorrelatedness-assumption is plausible (Hillebrand and Barnes, 2005); and iv) modified beamformer approaches are available to deal with those rare cases where sources with strong linear interactions are present (Brookes et al., 2007; Dalal et al., 2006; Diwakar et al., 2011; Hui et al., 2010; Quraan and Cheyne, 2010). In addition, the PLI and beamformer blindspots sit comfortably together — the beamformer will potentially mis-localise sources with zero-lag correlation but by using the PLI we ignore these effects.

Other methods, such as minimum norm based approaches, have no such constraints on source correlation. However, this flexibility comes at the price of poorer noise rejection capability. The work of Ghuman et al. highlights the problems that occur when combining a minimum norm approach with a *phase-locking* (rather than *phase-lagging*) approach, in that one has to rely on the subtraction of empty room data in order to try to remove the large number of spurious interactions (Ghuman et al., 2011). A particular worry here is that minimum norm based source reconstruction approaches inherently model all the data and therefore project artefacts (such as heartbeat or from external noise sources) into the source space. In contrast, beamforming approaches only localise those components in the data that match the lead field for a source at a particular location, giving beamformers the ability to reject artefacts (e.g. Adjamian et al., 2009). Importantly, these projected artefacts lead to further spurious connectivity estimates when using methods that are sensitive to zero-phase interactions (i.e. methods based on phase-locking, rather than PLI).

Beamformer implementations exist that use the matrix of connections (Gross et al., 2001) or higher order statistics (Huang et al., 2004), rather than the data covariance matrix, for the computation of the beamformer weights (Eq. (2)). One could also consider the direct replacement of the data covariance matrix by the PLI adjacency matrix when computing the beamformer weights, thereby avoiding problems related to volume conduction and zero-lag phase relations between sources. This may be problematic though, since the PLI is independent of signal amplitude. However, further research could show whether the use of the imaginary coherence, which also minimises the influence of volume conduction, proves to be more fruitful in this type of approach.

#### Selection of ROIs

We chose to parcellate the source space on the basis of the Talairach Daemon Database, although alternative atlases are available (e.g. Collins et al., 1995; Tzourio-Mazoyer et al., 2002). Instead, one could also use a parcellation-scheme that is based on the source-sensor geometry in order to obtain a set of maximally independent patches (Palva et al., 2010a), or perform parcellation in the native MRIs (Seibert and Brewer, 2011). This latter option would in particular be preferably for patients who's MRI match poorly to a template MRI, for example due to atrophy. In addition, it remains an open question how best to deal with ROIs of unequal size. One could argue that all ROIs should contain an equal number of voxels so that estimates of interdependencies are not affected by differences in ROI size. In contrast, perhaps the size of a ROI should reflect the variations in sensitivity of MEG to neuronal activity in different regions of the brain (Hillebrand and Barnes, 2002). Overall, these disadvantages of using a standard brain-atlas are outweighed by the important advantage that its use enables a more direct comparison between data from different modalities (Plis et al., 2011).

#### Beamformer weight estimation

Here, we used the beamformer formulation for point sources, i.e. the lead fields for equivalent current dipoles were used for the weight computations. In order to take into account the spatial extent of the ROIs for which representative time-series are estimated, one could use the lead fields for spatially extended sources (Hillebrand and Barnes, 2011), use a set of basis functions (Limpiti et al., 2004, 2006), or use Singular Value Decomposition (SVD) to define a lower-dimensional representation for each ROI (Gross and Ioannides, 1999). Similarly, a combination of Signal Space Separation (SSS) and beamforming allows for the estimation of time-series for pre-defined spherical ROIs (Ozkurt et al., 2009), although with this approach one would lose the advantages of using a standard brain atlas. Instead, the direct replacement of the SAM beamformer by the recently developed SSS-beamformer (Vrba et al., 2010) would fit more naturally in our proposed analysis framework.

Recently, Hui et al. (2010) have proposed a nulling-beamformer, which removes potential cross-talk between ROIs by incorporating additional (nulling-) constraints for these ROIs into the beamformer design. It is not clear though how the reduction in the degrees of freedom due to the use of many ROIs in a standard atlas (i.e. many nulling-constraints would be needed) would affect both the ability to reject noise and the accuracy of the reconstructed time-series.

We used a multi-sphere head model for the computation of the lead fields, which provides an accurate approximation of the volume conductor for MEG (Huang et al., 1999), although more complex numerical models may provide increased accuracy in certain situations (Lalancette et al., 2011). Inaccuracies in the volume conductor model may lead to underestimation of source power (Hillebrand and Barnes, 2003, 2005). However, given that PLI is independent of amplitude, it is unlikely that connectivity biases were introduced, even if there were regions for which source power was underestimated.

The length of the data covariance window that was used for the weight computations was determined by the amount of data that was available from each recording session, the duration of which was set at what was common practice for a resting-state MEG session at the time of recording (2003/2004). Simulation studies have shown that the required number of samples for beamforming depends, among other factors, on the frequency-band of interest, sampling rate and source power (Brookes et al., 2008). The results by Brookes et al. suggest that even for our worst case scenario (small band width of 3.5 Hz for the delta band and smallest co-variance window of 175 s) the errors in the estimation of the data covariance, and therefore in source power estimates, were minimal (less than 10% underestimation in source power).

It is feasible to apply noise-regularisation during the computation of the beamformer weights (not used in this study), which would lead to increased signal-to-noise ratio (SNR) for the estimated time-series, and would also mitigate against the effects of using a limited set of (atlas-based) voxels in template space (activation could be missed due to co-registration and normalisation errors — see e.g. Beal et al. (2010) for a similar strategy). However, regularisation comes at the expense of a decreased ability to distinguish spatially separate sources (Gross and Ioannides, 1999). The optimal trade-off between this temporal and spatial accuracy for studies that aim to compute the topography and topology of functional networks, has yet to be determined for empirical data.

#### Defining representative time-series for a ROI

To deal with the issue of arbitrary sign for the orientation of the source at each voxel, which makes straightforward averaging of source waveforms across a ROI impossible, we selected the voxel with maximum source power within a ROI. It has been shown previously (Barnes et al., 2004) that the time-series estimated at local maxima best describe the underlying source activity. For datasets with large artefacts, these artefacts could leak into the reconstructed time-series and potentially bias the selection of the voxel with maximum power. Alternative approaches include: i) performing a check on (and adjustment of) the polarity of the time-series of neighbouring voxels before averaging time-series across a ROI. This assumes that the source orientation varies smoothly when moving through the source space; ii) using SVD to find the eigenvectors (time-series) that best represent the time-series of the ROI (see e.g. Supp et al., 2007); and iii) using the time-series that most strongly correlates with the time-series for the other voxels in the ROI.

A consequence of selecting the voxels with maximum power to represent the ROIs is that, particularly when source activation spreads over multiple ROIs, the peak voxels for neighbouring ROIs can be close together (Supplementary Fig. 5), i.e. such voxels share (almost) the same signal. For such cases, the source reconstruction approach could not unambiguously determine whether the activity is coming from one or the other ROI (or both). Importantly, PLI is insensitive to the spurious zero-lag interactions that could exist between voxels that are close together, hence the voxel selection will not lead to overestimates of (local) connectivity.

Alternatively, one could, for each voxel, extract the power modulations of the time-series using the Hilbert transform (Byron and Fuller, 1992) and average these envelopes across a ROI. This approach has already demonstrated interesting relationships between functional networks constructed on the basis of fluctuations in MEG band-limited power and those based on low-frequency modulations in BOLD fMRI time-series (Liu et al., 2010). A limitation of this approach is that differences in ROI size might result in biases due to differences in SNR.

#### Connectivity estimation

In this work we contrasted PLI with Phase Coherence as measures of functional connectivity between ROIs. As expected, the Phase Coherence suffers from spurious correlations between ROIs due to correlations between their beamformer weights (Fig. 3). PLI on the other hand is a conservative measure that is insensitive to such spurious interactions, albeit at the expense that true zero-lag correlations are also missed. Zero-lag correlations are most-likely to be short-range (e.g. Gray et al., 1989), but see (Rodriguez et al., 1999; Roelfsema et al., 1997; Tognoli and Kelso, 2009; Vicente et al., 2008), and functional networks constructed on the basis of PLI could therefore have a topology for which the clustering and/or modularity are underestimated. Note that this is a general issue for all MEG/EEG connectivity studies, and not a problem that is specific for the proposed analysis framework. In fact, when improved connectivity estimators become available, then they can easily be incorporated in our analysis framework. Vinck et al. (2011) have recently described a promising modification of PLI with reduced underestimation of connectivity between sources with small-lag interactions, as well as a reduced estimator bias, although this comes at the expense of introducing an arbitrary bias favouring large phase differences and mixing of the estimation of consistency of phase differences with the estimation of the magnitude of the phase difference. An alternative approach that would avoid these issues, and at the same time reduce the effects of noise on PLI-based connectivity estimates (particularly when interactions occur with almost zero-phase lag) would be to ignore phase differences within a small window around zero and around ± π. However, although the use of such an offset could potentially be useful when considering small numbers of trials, noise-related counts of positive and negative phase lags (regardless of magnitude) should not bias the statistics.

We should note that the time window for the connectivity analysis was fixed here at 13.1 s, and that we analysed 5 of such artefact-free data-segments. Clearly, the choice of time window determines not only the expected period of stationarity of the interactions, but also the bandwidth over which interactions can occur. Further investigations may reveal whether different forms of interactions are highlighted for different choices of time-frequency parameters. Indeed, empirical observations have revealed that relatively short epochs (~ 10 s) are quasi-stationary, and that such short epochs can already be representative of a subject's cognitive fingerprint (see Schomer and Lopes da Silva, 2010). Moreover, the work by Honey et al. (2007) has shown that functional networks at these time-scales are stable yet dynamic (on longer time-scales the functional networks resemble the underlying (static) anatomical network, whereas on shorter timescales the functional networks are highly variable). Additionally, it has been shown that using 5 epochs of 10 s resting-state data results in fairly good to good levels of test–retest reliabilities, depending on the frequency band and metrics analysed (Jin et al., 2011). It should be noted that Lin et al. analysed magnetometer data at the sensor level and computed complex network metrics, whereas in our study third-order gradiometer data was analysed using a beamformer approach, both resulting in improved SNR, and that we used simple metrics to characterise the functional networks; these factors all contribute to an increased stability of the estimated resting-state network properties (Deuker et al., 2009; Jin et al., 2011). Finally, using the same number of epochs as we routinely use in our clinical studies, which has proven to give stable estimates of resting-state activity/network parameters (e.g. Douw et al., 2011), renders our developed methodology and the results from the current study directly relevant to our clinical work.

We looked at the average overall connectivity for a ROI, not, as is typical in fMRI, specifically at connections between certain ROIs. For example, the average connectivity for a ROI could be relatively low, but the ROI could still have strong connections with (only) some ROIs in, let's say, the default mode network. A more in depth comparison with the fMRI literature would be an interesting topic for future research. Indeed, recent work (Brookes et al., 2011b) has shown the correspondence between beamformer estimated power envelope correlations and fMRI defined resting-state networks. It will be interesting to see the make-up of the fMRI defined networks in terms of the relative contributions of electrical interactions over dimensions of power, phase and frequency.

#### Statistics

For the creation of the phase-randomised surrogates we did not take into account any jumps at the boundaries, which could have introduced high-frequency artefacts in our surrogate data (Kantz and Schreiber, 1997). However, any biases introduced in our statistics will have been minimal, as we subsequently filtered the surrogate data to relatively low frequencies (maximal 48 Hz for the gamma band).

We have introduced a general MEG analysis framework for the reconstruction of frequency-dependent profiles of source power and functional connectivity, which is robust to artefactual connectivity estimates caused by volume conduction, due to the use of the PLI; robust to artefactual connectivity estimates caused by physiological (e.g. heartbeat) and environmental (e.g. power-line) noise, due to the use of the beamformer approach; robust to co-registration errors, due to the use of unconstrained source orientation at each voxel; and robust to modelling errors (e.g. introduced by co-registration errors or by ignoring source extent), due to the use of coarse spatial sampling (defined by the ROIs). Finally, the results are based on non-parametric statistics with few underlying assumptions.

The analysis framework contains two important elements: i) activity is reconstructed for an atlas-based set of ROIs in order to facilitate interpretation and comparison with results obtained with other modalities; and ii) effects of volume conduction/field spread on estimated interactions between ROIs are removed using PLI, a measure that is insensitive to these effects. Using this framework we have revealed distinct frequency-dependent patterns of source power and source interactions. We envisage that this approach will be used to further elucidate the patterns of resting-state activity in health and disease (Fox and Greicius, 2010; Guggisberg et al., 2008; Martino et al., 2011; Ortega et al., 2008).

The following are the supplementary materials related to this article.

Supplementary Fig. 1 PLI-based adjacency matrices for delta, theta, alpha, beta and gamma bands. The separation between anatomical groupings (from left to right: occipital, parietal/central, temporal, frontal) is denoted by a solid line, the separation between left and right hemisphere within each anatomical grouping is denoted by a dotted line (see Appendix A for details).

Supplementary Fig. 2 Mean PLI (left column) and mean relative power (right column) for delta (upper row) and theta bands (bottom row), displayed as a colour-coded map (unthresholded) on a schematic of the parcellated template brain.

Supplementary Fig. 3 Mean thresholded PLI (left column) and mean relative power (right column) for alpha1 (upper row) and alpha2 (bottom row), displayed as a colour-coded map on a schematic of the parcellated template brain. Note that alpha1 band PLI did not reach significance.

Supplementary Fig. 4 Mean PLI (left column) and mean degree (using PLI values thresholded at 20% of maximum) (right column), for delta, theta, alpha, beta and gamma bands (top to bottom).

Supplementary Fig. 5 a) Distance between peak voxels (in cm) averaged over subjects and frequency bands. The separation between anatomical groupings (from left to right: occipital, parietal/central, temporal, frontal) is denoted by a solid line, the separation between left and right hemisphere within each anatomical grouping is denoted by a dotted line (see Appendix A for details). The average distance was small between same ROIs (minimum distance was 0.7 cm for left BA3 and BA4), suggesting that the source reconstruction approach could not unambiguously determine whether the activity came from one or the other ROI (or both); b) scatter plot of the (squared) correlation between beamformer weights and the mean distance between peak voxels. Note that larger average weight correlation is found for peak voxels that are closer together, as is expected due to the correlation between lead fields for such voxels.

## Figures and Tables

**Table 1:** List of the Brodmann areas, and the labels, that were used. L denotes left hemisphere, R denotes right hemisphere. For the display of the adjacency matrices, the following groupings were defined based on the indices in this table: left occipital (7, 9, 11), right occipital (8, 10, 12), left parietal/central (1, 13, 27, 59, 63, 39, 43, 49, 29, 21), right parietal/central (2, 14, 28, 60, 64, 40, 44, 50, 30, 22), left temporal (45, 47, 19, 17, 15, 35, 37), right temporal (46, 48, 20, 18, 16, 36, 38), left frontal (41, 61, 65, 67, 3, 55, 53, 51, 5, 57, 25, 31, 23, 33), right frontal (42, 62, 66, 68, 4, 56, 54, 52, 6, 58, 26, 32, 24, 34).

Index	ROI label	Index	ROI label
1	BA 1: primary somatosensory cortex (L)	35	BA 37: fusiform gyrus (L)
2	BA 1: primary somatosensory cortex (R)	36	BA 37: fusiform gyrus (R)
3	BA 10: anterior prefrontal cortex (L)	37	BA 38: temporopolar area (L)
4	BA 10: Anterior prefrontal cortex (R)	38	BA 38: temporopolar area (R)
5	BA 11: orbitofrontal cortex (L)	39	BA 39: angular gyrus (L)
6	BA 11: orbitofrontal cortex (R)	40	BA 39: angular gyrus (R)
7	BA 17: primary visual cortex (L)	41	BA 4: primary motor cortex (L)
8	BA 17: primary visual cortex (R)	42	BA 4: primary motor cortex (R)
9	BA 18: secondary visual cortex (L)	43	BA 40: supramarginal gyrus (L)
10	BA 18: secondary visual cortex (R)	44	BA 40: supramarginal gyrus (R)
11	BA 19: associative visual cortex (L)	45	BA 41: primary and auditory association cortex (L)
12	BA 19: associative visual cortex (R)	46	BA 41: primary and auditory association cortex (R)
13	BA 2: primary somatosensory cortex (L)	47	BA 42: primary and auditory association cortex (L)
14	BA 2: primary somatosensory cortex (R)	48	BA 42: primary and auditory association cortex (R)
15	BA 20: inferior temporal gyrus (L)	49	BA 43: primary gustatory cortex (L)
16	BA 20: inferior temporal gyrus (R)	50	BA 43: primary gustatory cortex (R)
17	BA 21: middle temporal gyrus (L)	51	BA 44: pars opercularis (L)
18	BA 21: middle temporal gyrus (R)	52	BA 44: pars opercularis (R)
19	BA 22: superior temporal gyrus (L)	53	BA 45: pars triangularis (L)
20	BA 22: superior temporal gyrus (R)	54	BA 45: pars triangularis (R)
21	BA 23: ventral posterior cingulate (L)	55	BA 46: dorsolateral prefrontal cortex (L)
22	BA 23: ventral posterior cingulate (R)	56	BA 46: dorsolateral prefrontal cortex (R)
23	BA 24: ventral anterior cingulate (L)	57	BA 47: inferior prefrontal gyrus (L)
24	BA 24: ventral anterior cingulate (R)	58	BA 47: inferior prefrontal gyrus(R)
25	BA 25: ventromedial prefrontal cortex (L)	59	BA 5: somatosensory association cortex (L)
26	BA 25: ventromedial prefrontal cortex (R)	60	BA 5: somatosensory association cortex (R)
27	BA 3: primary somatosensory cortex (L)	61	BA 6: premotor cortex and supplementary motor area (L)
28	BA 3: primary somatosensory cortex (R)	62	BA 6: premotor cortex and supplementary motor area (R)
29	BA 31: dorsal posterior cingulate cortex (L)	63	BA 7: somatosensory association cortex (L)
30	BA 31: dorsal posterior cingulate cortex (R)	64	BA 7: somatosensory association cortex (R)
31	BA 32: dorsal anterior cingulate cortex (L)	65	BA 8: frontal cortex including frontal eye fields (L)
32	BA 32: dorsal anterior cingulate cortex (R)	66	BA 8: frontal cortex including frontal eye fields (R)
33	BA 33: anterior cingulate cortex (L)	67	BA 9: dorsolateral prefrontal cortex (L)
34	BA 33: anterior cingulate cortex (R)	68	BA 9: dorsolateral prefrontal cortex (R)

**Table 2:** Ranked list of the Brodmann areas that had significant (*p* < 0.05) mean PLI with all other ROIs. BA denotes Brodmann area, L denotes left hemisphere, R denotes right hemisphere.

Alpha	Beta	Gamma
BA 31: dorsal posterior cingulate (R)	BA 4: primary motor (L)	BA 21: middle temporal gyrus (R)
BA 19: associative visual (R)	BA 40: supramarginal gyrus (L)	BA 3: primary somatosensory (R)
BA 23: ventral posterior cingulate (L)	BA 3: primary somatosensory (L)	BA 45: pars triangularis (R)
BA 23: ventral posterior cingulate (R)	BA 1: primary somatosensory (L)	BA 41: primary auditory (R)
BA 39: angular gyrus (R)	BA 4: primary motor (R)	BA 43: gustatory cortex (R)
BA 22: superior temporal gyrus (R)	BA 1: primary somatosensory (R)	BA 44: pars opercularis (R)
BA 18: secondary visual (R)	BA 6: secondary motor (R)	BA 2: primary somatosensory (R)
BA 17: primary visual (R)	BA 2: primary somatosensory (L)	BA 40: supramarginal gyrus (R)
BA 37: fusiform gyrus (R)	BA 3: primary somatosensory (R)	BA 4: primary motor (R)
BA 19: associative visual (L)	BA 6: secondary motor (L)	BA 42: primary auditory (R)
BA 7: somatosensory association (R)	BA 2: primary somatosensory (R)	BA 38: temporopolar area (R)
BA 37: fusiform gyrus (L)	BA 40: supramarginal gyrus (R)	BA 2: primary somatosensory (L)
BA 7: somatosensory association (L)	BA 5: somatosensory association (L)	BA 1: primary somatosensory (R)
BA 17: primary visual (L)	BA 31: dorsal posterior cingulate (L)	BA 6: secondary motor (R)
BA 18: secondary visual (L)		BA 20: inferior temporal gyrus (R)
BA 31: dorsal posterior cingulate (L)		BA 3: primary somatosensory (L)
		BA 9: dorsolateral prefrontal (L)
		BA 22: superior temporal gyrus (R)
		BA 1: primary somatosensory (L)

## Footnotes

1 The lead field is defined as the MEG sensor signal that is produced by a source of unitary strength.

## References

[bb0005] Adjamian P., Holliday I.E., Barnes G.R., Hillebrand A., Hadjipapas A., Singh K.D. (2004). Induced visual illusions and gamma oscillations in human primary visual cortex. Eur. J. Neurosci..

[bb0010] Adjamian P., Worthen S.F., Hillebrand A., Furlong P.L., Chizh B.A., Hobson A.R., Aziz Q., Barnes G.R. (2009). Effective electromagnetic noise cancellation with beamformers and synthetic gradiometry in shielded and partly shielded environments. J. Neurosci. Methods.

[bb0015] Altamura M., Goldberg T.E., Elvevag B., Holroyd T., Carver F.W., Weinberger D.R., Coppola R. (2010). Prefrontal cortex modulation during anticipation of working memory demands as revealed by magnetoencephalography. Int. J. Biomed. Imaging.

[bb0020] Arieli A., Sterkin A., Grinvald A., Aertsen A. (1996). Dynamics of ongoing activity: explanation of the large variability in evoked responses. Science.

[bb0025] Astolfi L., Cincotti F., Mattia D., Marciani M.G., Baccala L.A., de Vico F.F., Salinari S., Ursino M., Zavaglia M., Ding L., Edgar J.C., Miller G.A., He B., Babiloni F. (2007). Comparison of different cortical connectivity estimators for high-resolution EEG recordings. Hum. Brain Mapp..

[bb0030] Babiloni F., Cincotti F., Babiloni C., Carducci F., Mattia D., Astolfi L., Basilisco A., Rossini P.M., Ding L., Ni Y., Cheng J., Christine K., Sweeney J., He B. (2005). Estimation of the cortical functional connectivity with the multimodal integration of high-resolution EEG and fMRI data by directed transfer function. Neuroimage.

[bb0035] Barnes G.R., Hillebrand A. (2003). Statistical flattening of MEG beamformer images. Hum. Brain Mapp..

[bb0040] Barnes G.R., Hillebrand A., Fawcett I.P., Singh K.D. (2004). Realistic spatial sampling for MEG beamformer images. Hum. Brain Mapp..

[bb0045] Bassett D.S., Meyer-Lindenberg A., Achard S., Duke T., Bullmore E. (2006). Adaptive reconfiguration of fractal small-world human brain functional networks. Proc. Natl. Acad. Sci. U.S.A..

[bb0050] Beal D.S., Cheyne D.O., Gracco V.L., Quraan M.A., Taylor M.J., De Nil L.F. (2010). Auditory evoked fields to vocalization during passive listening and active generation in adults who stutter. Neuroimage.

[bb0055] Born R.T., Bradley D.C. (2005). Structure and function of visual area MT. Annu. Rev. Neurosci..

[bb0060] Bosboom J.L., Stoffers D., Stam C.J., van Dijk B.W., Verbunt J., Berendse H.W., Wolters E.C. (2006). Resting state oscillatory brain dynamics in Parkinson's disease: an MEG study. Clin. Neurophysiol..

[bb0065] Bosboom J.L., Stoffers D., Wolters E.C., Stam C.J., Berendse H.W. (2009). MEG resting state functional connectivity in Parkinson's disease related dementia. J. Neural Transm..

[bb0075] Brookes M.J., Stevenson C.M., Barnes G.R., Hillebrand A., Simpson M.I., Francis S.T., Morris P.G. (2007). Beamformer reconstruction of correlated sources using a modified source model. Neuroimage.

[bb0755] Brookes M.J., Vrba J., Robinson S.E., Stevenson C.M., Peters A.M., Barnes G.R., Hillebrand A., Morris P.G. (2008). Optimising experimental design for MEG beamformer imaging. Neuroimage.

[bb0070] Brookes M.J., Hale J.R., Zumer J.M., Stevenson C.M., Francis S.T., Barnes G.R., Owen J.P., Morris P.G., Nagarajan S.S. (2011). Measuring functional connectivity using MEG: methodology and comparison with fcMRI. Neuroimage.

[bb0080] Brookes M.J., Woolrich M., Luckhoo H., Price D., Hale J.R., Stephenson M.C., Barnes G.R., Smith S.M., Morris P.G. (2011). Investigating the electrophysiological basis of resting state networks using magnetoencephalography. Proc. Natl. Acad. Sci. U.S.A..

[bb0085] Byron F.W., Fuller R.W. (1992). Mathematics of Classical and Quantum Physics.

[bb0090] Caramia M.D., Palmieri M.G., Giacomini P., Iani C., Dally L., Silvestrini M. (2000). Ipsilateral activation of the unaffected motor cortex in patients with hemiparetic stroke. Clin. Neurophysiol..

[bb0095] Chawla D., Lumer E.D., Friston K.J. (1999). The relationship between synchronization among neuronal populations and their mean activity levels. Neural Comput..

[bb0100] Chawla D., Lumer E.D., Friston K.J. (2000). Relating macroscopic measures of brain activity to fast, dynamic neuronal interactions. Neural Comput..

[bb0105] Chen A.C., Feng W., Zhao H., Yin Y., Wang P. (2008). EEG default mode network in the human brain: spectral regional field powers. Neuroimage.

[bb0110] Cheung B.L., Riedner B.A., Tononi G., van Veen B.D. (2010). Estimation of cortical connectivity from EEG using state-space models. IEEE Trans. Biomed. Eng..

[bb0115] Cheyne D., Bostan A.C., Gaetz W., Pang E.W. (2007). Event-related beamforming: a robust method for presurgical functional mapping using MEG. Clin. Neurophysiol..

[bb0120] Collins D.L., Holmes C.J., Peters T.M., Evans A.C. (1995). Automatic 3-D model-based neuroanatomical segmentation. Hum. Brain Mapp..

[bb0125] Congedo M., John R.E., De R.D., Prichep L. (2010). Group independent component analysis of resting state EEG in large normative samples. Int. J. Psychophysiol..

[bb0130] Daffertshofer A., van Wijk B.C. (2011). On the influence of amplitude on the connectivity between phases. Front Neuroinform..

[bb0135] Dalal S.S., Sekihara K., Nagarajan S.S. (2006). Modified beamformers for coherent source region suppression. IEEE Trans. Biomed. Eng..

[bb0140] David O., Garnero L., Cosmelli D., Varela F. (2002). Estimation of neural dynamics from MEG/EEG cortical current density maps: application to the reconstruction of large-scale cortical synchrony. IEEE Trans. Biomed. Eng..

[bb0145] David O., Cosmelli D., Lachaux J.P., Baillet S., Garnero L., Martinerie J. (2003). A theoretical and experimental introduction to the non-invasive study of large-scale neural phase synchronization in human beings. Int. J. Comput. Cogn..

[bb0150] de Pasquale F., Della Penna S., Snyder A.Z., Lewis C., Mantini D., Marzetti L., Belardinelli P., Ciancetta L., Pizzella V., Romani G.L., Corbetta M. (2010). Temporal dynamics of spontaneous MEG activity in brain networks. Proc. Natl. Acad. Sci. U.S.A..

[bb0155] De Vico Fallani F., Astolfi L., Cincotti F., Mattia D., Marciani M.G., Salinari S., Kurths J., Gao S., Cichocki A., Colosimo A., Babiloni F. (2007). Cortical functional connectivity networks in normal and spinal cord injured patients: evaluation by graph analysis. Hum. Brain Mapp..

[bb0160] Deuker L., Bullmore E.T., Smith M., Christensen S., Nathan P.J., Rockstroh B., Bassett D.S. (2009). Reproducibility of graph metrics of human brain functional networks. Neuroimage.

[bb0165] Ding L., Worrell G.A., Lagerlund T.D., He B. (2007). Ictal source analysis: localization and imaging of causal interactions in humans. Neuroimage.

[bb0170] Diwakar M., Tal O., Liu T.T., Harrington D.L., Srinivasan R., Muzzatti L., Song T., Theilmann R.J., Lee R.R., Huang M.X. (2011). Accurate reconstruction of temporal correlation for neuronal sources using the enhanced dual-core MEG beamformer. Neuroimage.

[bb0175] Domínguez L.G., Wennberg R., Velázquez J.L.P., Erra R.G. (2007). Enhanced measured synchronization of unsynchronized sources: inspecting the physiological significance of synchronization analysis of whole brain electrophysiological recordings. Int. J. Phys. Sci..

[bb0180] Dossevi A., Cosmelli D., Garnero L., Ammari H. (2008). Multivariate reconstruction of functional networks from cortical sources dynamics in MEG/EEG. IEEE Trans. Biomed. Eng..

[bb0185] Douw L., Schoonheim M.M., Landi D., van der Meer M.L., Geurts J.J., Reijneveld J.C., Klein M., Stam C.J. (2011). Cognition is related to resting-state small-world network topology: an magnetoencephalographic study. Neuroscience.

[bb0190] Engel A.K., Fries P., Singer W. (2001). Dynamic predictions: oscillations and synchrony in top-down processing. Nat. Rev. Neurosci..

[bb0195] Farmer S.F. (1998). Rhythmicity, synchronization and binding in human and primate motor systems. J. Physiol..

[bb0200] Forss N., Silen T. (2001). Temporal organization of cerebral events: neuromagnetic studies of the sensorimotor system. Rev. Neurol. (Paris).

[bb0205] Fox M.D., Greicius M. (2010). Clinical applications of resting state functional connectivity. Front. Syst. Neurosci..

[bb0210] Fries P. (2005). A mechanism for cognitive dynamics: neuronal communication through neuronal coherence. Trends Cogn. Sci..

[bb0215] Friston K.J., Holmes A.P., Worsley K.J., Poline J.B., Frith C.D., Frackowiak R.S.J. (1995). Statistical parametric maps in functional imaging: a general linear approach. Hum. Brain Mapp..

[bb0220] Friston K.J., Li B., Daunizeau J., Stephan K.E. (2011). Network discovery with DCM. Neuroimage.

[bb0225] Gaetz W., Cheyne D. (2006). Localization of sensorimotor cortical rhythms induced by tactile stimulation using spatially filtered MEG. Neuroimage.

[bb0230] Ghuman A.S., McDaniel J.R., Martin A. (2011). A wavelet-based method for measuring the oscillatory dynamics of resting-state functional connectivity in MEG. Neuroimage.

[bb0235] Gomez-Herrero G., Atienza M., Egiazarian K., Cantero J.L. (2008). Measuring directional coupling between EEG sources. Neuroimage.

[bb0240] Gong G., He Y., Concha L., Lebel C., Gross D.W., Evans A.C., Beaulieu C. (2009). Mapping anatomical connectivity patterns of human cerebral cortex using in vivo diffusion tensor imaging tractography. Cereb. Cortex.

[bb0245] Grasman R.P., Huizenga H.M., Waldorp L.J., Bocker K.B., Molenaar P.C. (2004). Frequency domain simultaneous source and source coherence estimation with an application to MEG. IEEE Trans. Biomed. Eng..

[bb0250] Gray C.M., König P., Engel A.K., Singer W. (1989). Oscillatory responses in cat visual cortex exhibit inter-columnar synchronization which reflects global stimulus properties. Nature.

[bb0255] Griffiths T.D., Kumar S., Sedley W., Nourski K.V., Kawasaki H., Oya H., Patterson R.D., Brugge J.F., Howard M.A. (2010). Direct recordings of pitch responses from human auditory cortex. Curr. Biol..

[bb0260] Grodzinsky Y. (2000). The neurology of syntax: language use without Broca's area. Behav. Brain Sci..

[bb0265] Gross J., Ioannides A.A. (1999). Linear transformations of data space in MEG. Phys. Med. Biol..

[bb0270] Gross J., Kujala J., Hämäläinen M., Timmermann L., Schnitzler A., Salmelin R. (2001). Dynamic imaging of coherent sources: studying neural interactions in the human brain. PNAS.

[bb0275] Gruber T., Trujillo-Barreto N.J., Giabbiconi C.M., Valdes-Sosa P.A., Muller M.M. (2006). Brain electrical tomography (BET) analysis of induced gamma band responses during a simple object recognition task. Neuroimage.

[bb0280] Guggisberg A.G., Honma S.M., Findlay A.M., Dalal S.S., Kirsch H.E., Berger M.S., Nagarajan S.S. (2008). Mapping functional connectivity in patients with brain lesions. Ann. Neurol..

[bb0285] Hadjipapas A., Hillebrand A., Holliday I.E., Singh K.D., Barnes G.R. (2005). Assessing interactions of linear and nonlinear neuronal sources using MEG beamformers: a proof of concept. Clin. Neurophysiol..

[bb0290] Hall S.D., Holliday I.E., Hillebrand A., Furlong P.L., Singh K.D., Barnes G.R. (2005). Distinct contrast response functions in striate and extra-striate regions of visual cortex revealed with magnetoencephalography (MEG). Clin. Neurophysiol..

[bb0295] Hall S.D., Holliday I.E., Hillebrand A., Singh K.D., Furlong P.L., Hadjipapas A., Barnes G.R. (2005). The missing link: analogous human and primate cortical gamma oscillations. Neuroimage.

[bb0300] Hari R., Salmelin R. (1997). Human cortical oscillations: a neuromagnetic view through the skull. Trends Neurosci..

[bb0305] Harle M., Rockstroh B.S., Keil A., Wienbruch C., Elbert T.R. (2004). Mapping the brain's orchestration during speech comprehension: task-specific facilitation of regional synchrony in neural networks. BMC Neurosci..

[bb0310] Haufe S., Tomioka R., Nolte G., Muller K.R., Kawanabe M. (2010). Modeling sparse connectivity between underlying brain sources for EEG/MEG. IEEE Trans. Biomed. Eng..

[bb0315] Hillebrand A., Barnes G.R. (2002). A quantitative assessment of the sensitivity of whole-head meg to activity in the adult human cortex. Neuroimage.

[bb0330] Hillebrand A., Barnes G.R. (2003). The use of anatomical constraints with MEG beamformers. Neuroimage.

[bb0325] Hillebrand A., Barnes G.R. (2005). Beamformer analysis of MEG data. Int. Rev. Neurobiol. (Spec. Vol. Magnetoencephalogr.).

[bb0320] Hillebrand A., Barnes G.R. (2011). Practical constraints on estimation of source extent with MEG beamformers. Neuroimage.

[bb0335] Hillebrand A., Singh K.D., Holliday I.E., Furlong P.L., Barnes G.R. (2005). A new approach to neuroimaging with magnetoencephalography. Hum. Brain Mapp..

[bb0340] Hinkley L.B., Owen J.P., Fisher M., Findlay A.M., Vinogradov S., Nagarajan S.S. (2010). Cognitive impairments in schizophrenia as assessed through activation and connectivity measures of magnetoencephalography (MEG) data. Front. Hum. Neurosci..

[bb0345] Hipp J.F., Engel A.K., Siegel M. (2011). Oscillatory synchronization in large-scale cortical networks predicts perception. Neuron.

[bb0350] Hoechstetter K., Bornfleth H., Weckesser D., Ille N., Berg P., Scherg M. (2004). BESA source coherence: a new method to study cortical oscillatory coupling. Brain Topogr..

[bb0355] Honey C.J., Kotter R., Breakspear M., Sporns O. (2007). Network structure of cerebral cortex shapes functional connectivity on multiple time scales. Proc. Natl. Acad. Sci. U.S.A..

[bb0360] Hoogenboom N., Schoffelen J.M., Oostenveld R., Parkes L.M., Fries P. (2006). Localizing human visual gamma-band activity in frequency, time and space. Neuroimage.

[bb0365] Houweling S., Beek P.J., Daffertshofer A. (2010). Spectral changes of interhemispheric crosstalk during movement instabilities. Cereb. Cortex.

[bb0375] Huang M.X., Mosher J.C., Leahy R.M. (1999). A sensor-weighted overlapping-sphere head model and exhaustive head model comparison for MEG. Phys. Med. Biol..

[bb0370] Huang M.X., Shih J.J., Lee R.R., Harrington D.L., Thoma R.J., Weisend M.P., Hanlon F., Paulson K.M., Li T., Martin K., Miller G.A., Canive J.M. (2004). Commonalities and differences among vectorized beamformers in electromagnetic source imaging. Brain Topogr..

[bb0380] Hui H.B., Pantazis D., Bressler S.L., Leahy R.M. (2010). Identifying true cortical interactions in MEG using the nulling beamformer. Neuroimage.

[bb0385] Hyvarinen A., Ramkumar P., Parkkonen L., Hari R. (2010). Independent component analysis of short-time Fourier transforms for spontaneous EEG/MEG analysis. Neuroimage.

[bb0390] Ioannides A.A., Kostopoulos G.K., Laskaris N.A., Liu L., Shibata T., Schellens M., Poghosyan V., Khurshudyan A. (2002). Timing and connectivity in the human somatosensory cortex from single trial mass electrical activity. Hum. Brain Mapp..

[bb0395] Jerbi K., Lachaux J.P., N'Diaye K., Pantazis D., Leahy R.M., Garnero L., Baillet S. (2007). Coherent neural representation of hand speed in humans revealed by MEG imaging. Proc. Natl. Acad. Sci. U.S.A..

[bb0400] Jin S.H., Seol J., Kim J.S., Chung C.K. (2011). How reliable are the functional connectivity networks of MEG in resting states?. J. Neurophysiol..

[bb0405] Jurkiewicz M.T., Gaetz W.C., Bostan A.C., Cheyne D. (2006). Post-movement beta rebound is generated in motor cortex: evidence from neuromagnetic recordings. Neuroimage.

[bb0410] Kantz H., Schreiber T. (1997). Nonlinear Time Series Analysis.

[bb0415] Kenet T., Bibitchkov D., Tsodyks M., Grinvald A., Arieli A. (2003). Spontaneously emerging cortical representations of visual attributes. Nature.

[bb0425] Kujala J., Pammer K., Cornelissen P., Roebroeck A., Formisano E., Salmelin R. (2006). Phase coupling in a cerebro-cerebellar network at 8–13 Hz during reading. Cereb. Cortex.

[bb0420] Kujala J., Gross J., Salmelin R. (2008). Localization of correlated network activity at the cortical level with MEG. Neuroimage.

[bb0430] Lalancette M., Quraan M., Cheyne D. (2011). Evaluation of multiple-sphere head models for MEG source localization. Phys. Med. Biol..

[bb0435] Lancaster J.L., Rainey L.H., Summerlin J.L., Freitas C.S., Fox P.T., Evans A.C., Toga A.W., Mazziotta J.C. (1997). Automated labeling of the human brain: a preliminary report on the development and evaluation of a forward-transform method. Hum. Brain Mapp..

[bb0440] Lancaster J.L., Woldorff M.G., Parsons L.M., Liotti M., Freitas C.S., Rainey L., Kochunov P.V., Nickerson D., Mikiten S.A., Fox P.T. (2000). Automated Talairach atlas labels for functional brain mapping. Hum. Brain Mapp..

[bb0445] Langheim F.J., Leuthold A.C., Georgopoulos A.P. (2006). Synchronous dynamic brain networks revealed by magnetoencephalography. Proc. Natl. Acad. Sci. U.S.A..

[bb0450] Leopold D.A., Logothetis N.K. (2003). Spatial patterns of spontaneous local field activity in the monkey visual cortex. Rev. Neurosci..

[bb0455] Limpiti T., van Veen B.D., Nowak R.D., Wakai R.T. (2004). Linearly constrained minimum variance source imaging using cortical bases. Neurol. Clin. Neurophysiol..

[bb0460] Limpiti T., van Veen B.D., Wakai R.T. (2006). Cortical patch basis model for spatially extended neural activity. IEEE Trans. Biomed. Eng..

[bb0465] Lin F.H., Witzel T., Hamalainen M.S., Dale A.M., Belliveau J.W., Stufflebeam S.M. (2004). Spectral spatiotemporal imaging of cortical oscillations and interactions in the human brain. Neuroimage.

[bb0470] Linkenkaer-Hansen K., Nikulin V.V., Palva S., Ilmoniemi R.J., Palva J.M. (2004). Prestimulus oscillations enhance psychophysical performance in humans. J. Neurosci..

[bb0475] Liu Z., Fukunaga M., de Zwart J.A., Duyn J.H. (2010). Large-scale spontaneous fluctuations and correlations in brain electrical activity observed with magnetoencephalography. Neuroimage.

[bb0480] Mantini D., Della Penna S., Marzetti L., de Pasquale F., Pizzella V., Corbetta M., Romani G.L. (2011). A signal processing pipeline for MEG resting state networks. Brain Connect..

[bb0485] Maratos F.A., Anderson S.J., Hillebrand A., Singh K.D., Barnes G.R. (2007). The spatial distribution and temporal dynamics of brain regions activated during the perception of object and non-object patterns. Neuroimage.

[bb0490] Mardia K.V. (1972). Statistics of Directional Data.

[bb0495] Martino J., Honma S.M., Findlay A.M., Guggisberg A.G., Owen J.P., Kirsch H.E., Berger M.S., Nagarajan S.S. (2011). Resting functional connectivity in patients with brain tumors in eloquent areas. Ann. Neurol..

[bb0500] Marzetti L., Del G.C., Nolte G. (2008). Understanding brain connectivity from EEG data by identifying systems composed of interacting sources. Neuroimage.

[bb0505] Meinecke F.C., Ziehe A., Kurths J., Muller K.R. (2005). Measuring phase synchronization of superimposed signals. Phys. Rev. Lett..

[bb0510] Mima T., Steger J., Schulman A.E., Gerloff C., Hallett M. (2000). Electroencephalographic measurement of motor cortex control of muscle activity in humans. Clin. Neurophysiol..

[bb0515] Moran R.J., Stephan K.E., Seidenbecher T., Pape H.C., Dolan R.J., Friston K.J. (2009). Dynamic causal models of steady-state responses. Neuroimage.

[bb0520] Mormann F., Lehnertz K., David P., Elger C.E. (2000). Mean phase coherence as a measure for phase synchronization and its application to the EEG of epilepsy patients. Phys. D.

[bb0525] Mosher J.C., Baillet S., Leahy R.M. (2003). Equivalence of linear approaches in bioelectromagnetic inverse solutions. IEEE Workshop on Statistical Signal Processing. Sep 28–Oct 1, 2003, St. Louis, Missouri, U.S.A..

[bb0530] Nolte G., Bai O., Wheaton L., Mari Z., Vorbach S., Hallett M. (2004). Identifying true brain interaction from EEG data using the imaginary part of coherency. Clin. Neurophysiol..

[bb0535] Nolte G., Marzetti L., Valdes S.P. (2009). Minimum Overlap Component Analysis (MOCA) of EEG/MEG data for more than two sources. J. Neurosci. Methods.

[bb0540] Nunez P.L., Srinivasan R., Westdorp A.F., Wijesinghe R.S., Tucker D.M., Silberstein R.B., Cadusch P.J. (1997). EEG coherency. I: statistics, reference electrode, volume conduction, Laplacians, cortical imaging, and interpretation at multiple scales. Electroencephalogr Clin. Neurophysiol..

[bb0545] Ortega G.J., Sola R.G., Pastor J. (2008). Complex network analysis of human ECoG data. Neurosci. Lett..

[bb0550] Ozkurt T.E., Sun M., Jia W., Sclabassi R.J. (2009). Spatial filtering of MEG signals for user-specified spherical regions. IEEE Trans. Biomed. Eng..

[bb0555] Palva J.M., Monto S., Kulashekhar S., Palva S. (2010). Neuronal synchrony reveals working memory networks and predicts individual memory capacity. Proc. Natl. Acad. Sci. U.S.A..

[bb0560] Palva S., Monto S., Palva J.M. (2010). Graph properties of synchronized cortical networks during visual working memory maintenance. Neuroimage.

[bb0565] Pereda E., Quiroga R.Q., Bhattacharya J. (2005). Nonlinear multivariate analysis of neurophysiological signals. Prog. Neurobiol..

[bb0570] Pfurtscheller G. (1992). Event-related synchronization (ERS): an electrophysiological correlate of cortical areas at rest. Electroencephalogr. Clin. Neurophysiol..

[bb0575] Plis S.M., Weisend M.P., Damaraju E., Eichele T., Mayer A., Clark V.P., Lane T., Calhoun V.D. (2011). Effective connectivity analysis of fMRI and MEG data collected under identical paradigms. Comput. Biol. Med..

[bb0580] Quraan M.A., Cheyne D. (2010). Reconstruction of correlated brain activity with adaptive spatial filters in MEG. Neuroimage.

[bb0585] Robinson S.E., Vrba J., Yoshimoto T., Kotani M., Kuriki S., Karibe H., Nakasato N. (1999). Functional neuroimaging by synthetic aperture magnetometry (SAM). Recent Advances in Biomagnetism.

[bb0590] Rodriguez E., George N., Lachaux J.P., Martinerie J., Renault B., Varela F.J. (1999). Perception's shadow: long-distance synchronization of human brain activity. Nature.

[bb0595] Roelfsema P.R., Engel A.K., Konig P., Singer W. (1997). Visuomotor integration is associated with zero time-lag synchronization among cortical areas. Nature.

[bb0600] Rosanova M., Casali A., Bellina V., Resta F., Mariotti M., Massimini M. (2009). Natural frequencies of human corticothalamic circuits. J. Neurosci..

[bb0605] Rubinov M., Sporns O. (2010). Complex network measures of brain connectivity: uses and interpretations. Neuroimage.

[bb0610] Schoffelen J.M., Gross J. (2009). Source connectivity analysis with MEG and EEG. Hum. Brain Mapp..

[bb0615] Schoffelen J.M., Gross J. (2011). Improving the interpretability of all-to-all pairwise source connectivity analysis in MEG with nonhomogeneous smoothing. Hum. Brain Mapp..

[bb0620] Schomer D.L., Lopes da Silva F.H. (2010). Niedermeyer's Electroencephalography: Basic Principles, Clinical Applications, and Related Fields.

[bb0625] Sedley W., Teki S., Kumar S., Overath T., Barnes G.R., Griffiths T.D. (2012). Gamma band pitch responses in human auditory cortex measured with magnetoencephalography. Neuroimage.

[bb0630] Seibert T.M., Brewer J.B. (2011). Default network correlations analyzed on native surfaces. J. Neurosci. Methods.

[bb0640] Sekihara K., Nagarajan S.S., Poeppel D., Marantz A., Miyashita Y. (2001). Reconstructing spatio-temporal activities of neural sources using an MEG vector beamformer technique. IEEE Trans. Biomed. Eng..

[bb0635] Sekihara K., Nagarajan S.S., Poeppel D., Marantz A. (2004). Asymptotic SNR of scalar and vector minimum-variance beamformers for neuromagnetic source reconstruction. IEEE Trans. Biomed. Eng..

[bb0645] Siegel M., Donner T.H., Oostenveld R., Fries P., Engel A.K. (2008). Neuronal synchronization along the dorsal visual pathway reflects the focus of spatial attention. Neuron.

[bb0650] Srinivasan R., Winter W.R., Nunez P.L. (2006). Source analysis of EEG oscillations using high-resolution EEG and MEG. Prog. Brain Res..

[bb0655] Stam C.J., Nolte G., Daffertshofer A. (2007). Phase lag index: assessment of functional connectivity from multi channel EEG and MEG with diminished bias from common sources. Hum. Brain Mapp..

[bb0660] Supp G.G., Schlogl A., Trujillo-Barreto N., Muller M.M., Gruber T. (2007). Directed cortical information flow during human object recognition: analyzing induced EEG gamma-band responses in brain's source space. PLoS One.

[bb0665] Taniguchi M., Kato A., Fujita N., Hirata M., Tanaka H., Kihara T., Ninomiya H., Hirabuki N., Nakamura H., Robinson S.E., Cheyne D., Yoshimine T. (2000). Movement-related desynchronization of the cerebral cortex studied with spatially filtered magnetoencephalography. Neuroimage.

[bb0670] Tognoli E., Kelso J.A. (2009). Brain coordination dynamics: true and false faces of phase synchrony and metastability. Prog. Neurobiol..

[bb0675] Tononi G., Edelman G.M. (1998). Consciousness and complexity. Science.

[bb0680] Tononi G., Edelman G.M., Sporns O. (1998). Complexity and coherency: integrating information in the brain. Trends Cogn. Sci..

[bb0685] Tzourio-Mazoyer N., Landeau B., Papathanassiou D., Crivello F., Etard O., Delcroix N., Mazoyer B., Joliot M. (2002). Automated anatomical labeling of activations in SPM using a macroscopic anatomical parcellation of the MNI MRI single-subject brain. Neuroimage.

[bb0690] Uhlhaas P.J., Singer W. (2006). Neural synchrony in brain disorders: relevance for cognitive dysfunctions and pathophysiology. Neuron.

[bb0695] van den Heuvel M.P., Hulshoff Pol H.E. (2010). Exploring the brain network: a review on resting-state fMRI functional connectivity. Eur. Neuropsychopharmacol..

[bb0700] van der Werf Y.D., Paus T. (2006). The neural response to transcranial magnetic stimulation of the human motor cortex. I. Intracortical and cortico-cortical contributions. Exp. Brain Res..

[bb0705] van der Werf Y.D., Sadikot A.F., Strafella A.P., Paus T. (2006). The neural response to transcranial magnetic stimulation of the human motor cortex. II. Thalamocortical contributions. Exp. Brain Res..

[bb0710] van Drongelen W., Yuchtman M., van Veen B.D., van Huffelen A.C. (1996). A spatial filtering technique to detect and localize multiple sources in the brain. Brain Topogr..

[bb0715] van Veen B.D., van Drongelen W., Yuchtman M., Suzuki A. (1997). Localization of brain electrical activity via linearly constrained minimum variance spatial filtering. IEEE Trans. Biomed. Eng..

[bb0720] Varela F., Lachaux J.-P., Rodriguez E., Martinerie J. (2001). The brainweb: phase synchronization and large-scale integration. Nat. Rev. Neurosci..

[bb0725] Vicente R., Gollo L.L., Mirasso C.R., Fischer I., Pipa G. (2008). Dynamical relaying can yield zero time lag neuronal synchrony despite long conduction delays. Proc. Natl. Acad. Sci. U.S.A..

[bb0730] Vinck M., Oostenveld R., van W.M., Battaglia F., Pennartz C.M. (2011). An improved index of phase-synchronization for electrophysiological data in the presence of volume-conduction, noise and sample-size bias. Neuroimage.

[bb0735] Vrba J., Robinson S.E. (2002). SQUID sensor array configurations for magnetoencephalography applications. Supercond. Sci. Technol..

[bb0740] Vrba J., Taulu S., Nenonen J., Ahonen A. (2010). Signal space separation beamformer. Brain Topogr..

[bb0745] Ward L.M. (2003). Synchronous neural oscillations and cognitive processes. Trends Cogn. Sci..

[bb0750] Wibral M., Rahm B., Rieder M., Lindner M., Vicente R., Kaiser J. (2011). Transfer entropy in magnetoencephalographic data: quantifying information flow in cortical and cerebellar networks. Prog. Biophys. Mol. Biol..

