# Supplementary material for: Frequency-dependent functional connectivity within resting-state networks: An atlas-based MEG beamformer solution
Source: Neuroimage. 2012 Feb 15;59(4-2):3909–21. doi: 10.1016/j.neuroimage.2011.11.005 (PMC3382730; doi:10.1016/j.neuroimage.2011.11.005)
Supplement: Supplementary Fig. 1 — PLI-based adjacency matrices for delta, theta, alpha, beta and gamma bands. The separation between anatomical groupings (from left to right: occipital, parietal/central, temporal, frontal) is denoted by a solid line, the separation between left and right hemisphere within each anatomical grouping is denoted by a dotted line (see Appendix A for details). [file mmc1.doc]

*Adjacency matrices*

The PLI-based adjacency matrices for the different frequency bands are displayed in Supplementary Figure 1. Note that a clear structure is more readily observable for the alpha, beta and gamma bands, suggesting that some ROIs, or groups of ROIs, are much stronger connected than others for these frequency bands. In contrast, the adjacency matrices for the delta and theta band appear less structured. Interestingly though, the topographic maps of average PLI do reveal clearly distinct (although more diffuse than for the alpha and beta bands) patterns of connectivity that show similarities with the patterns of relative power for these frequency bands (Supplementary Figure 2). This further illustrates that graph analysis is required to fully (quantitatively) characterise the topology of these networks of functional connectivity (an example of which is given in Supplementary Figure 4).

| *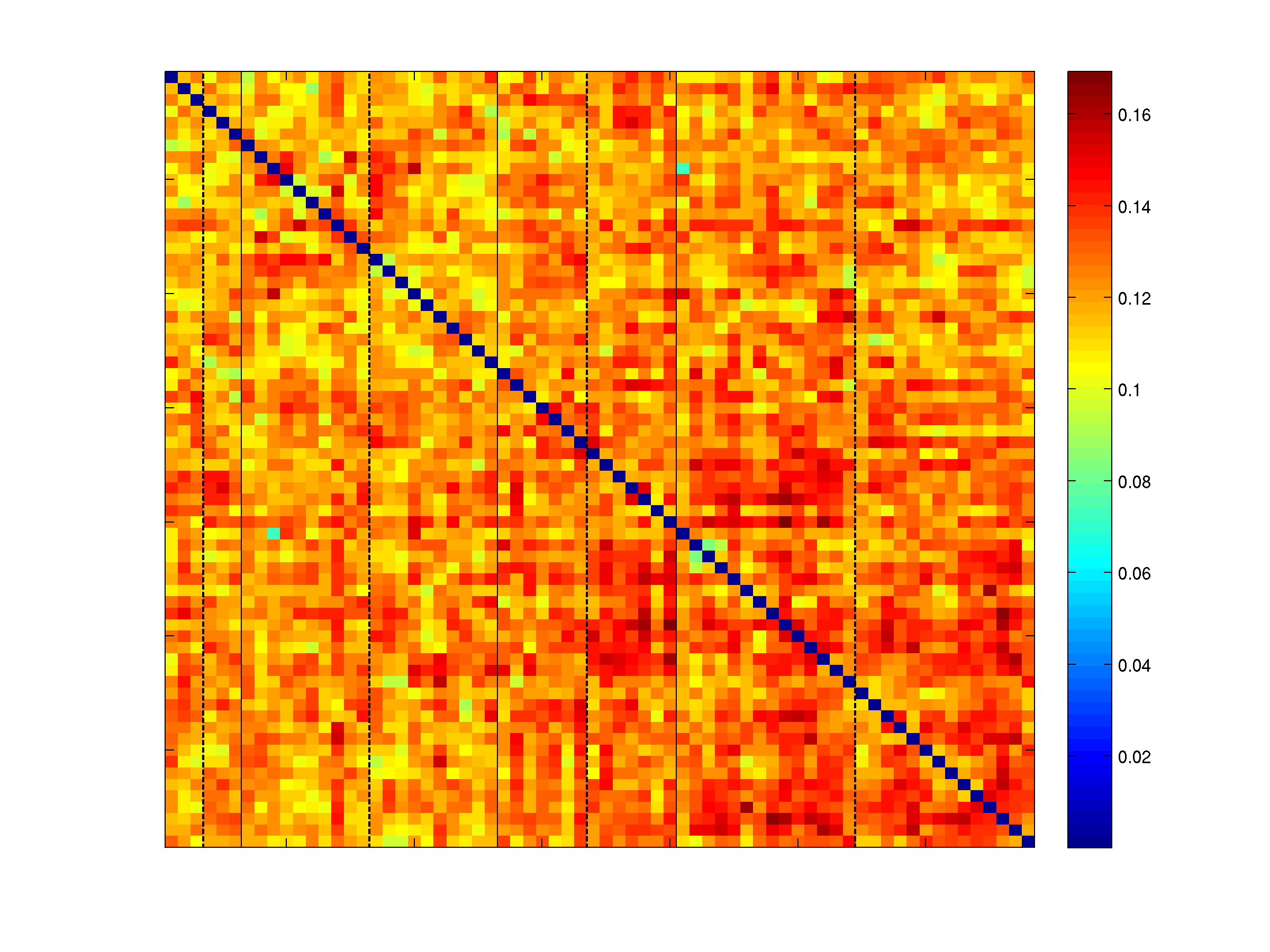*  **δ** | *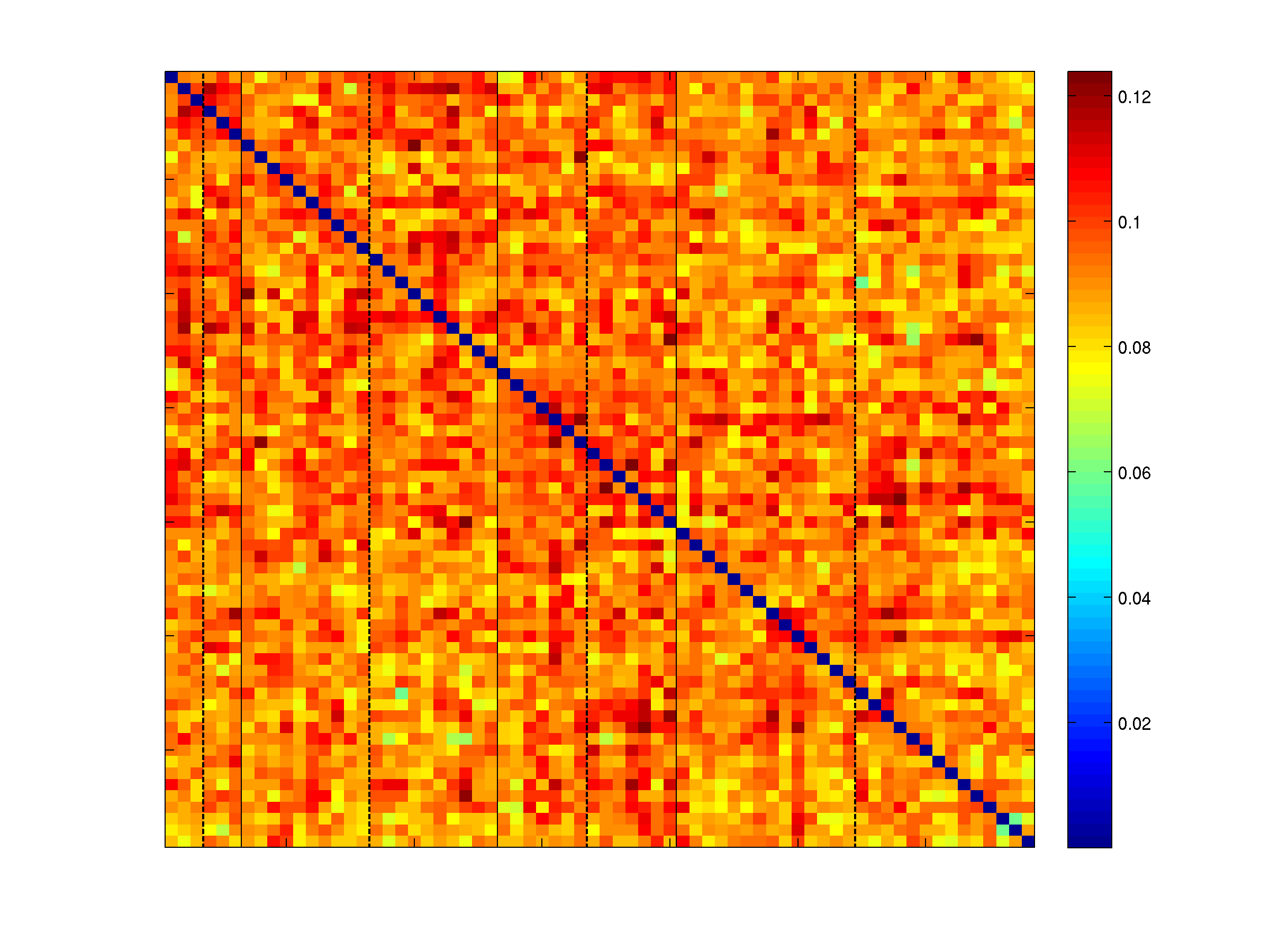*  **θ** | *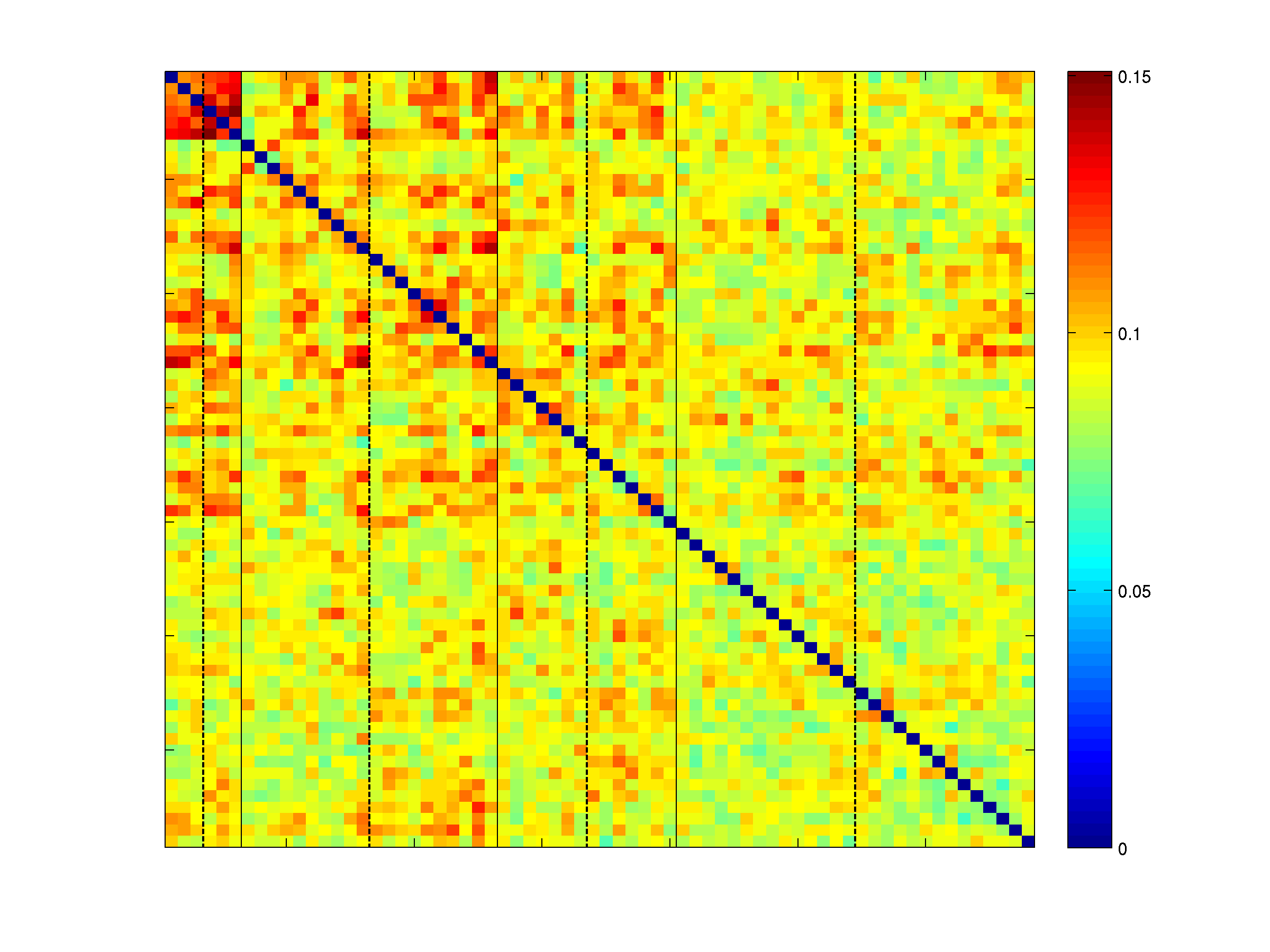*  **α** |
| --- | --- | --- |
| *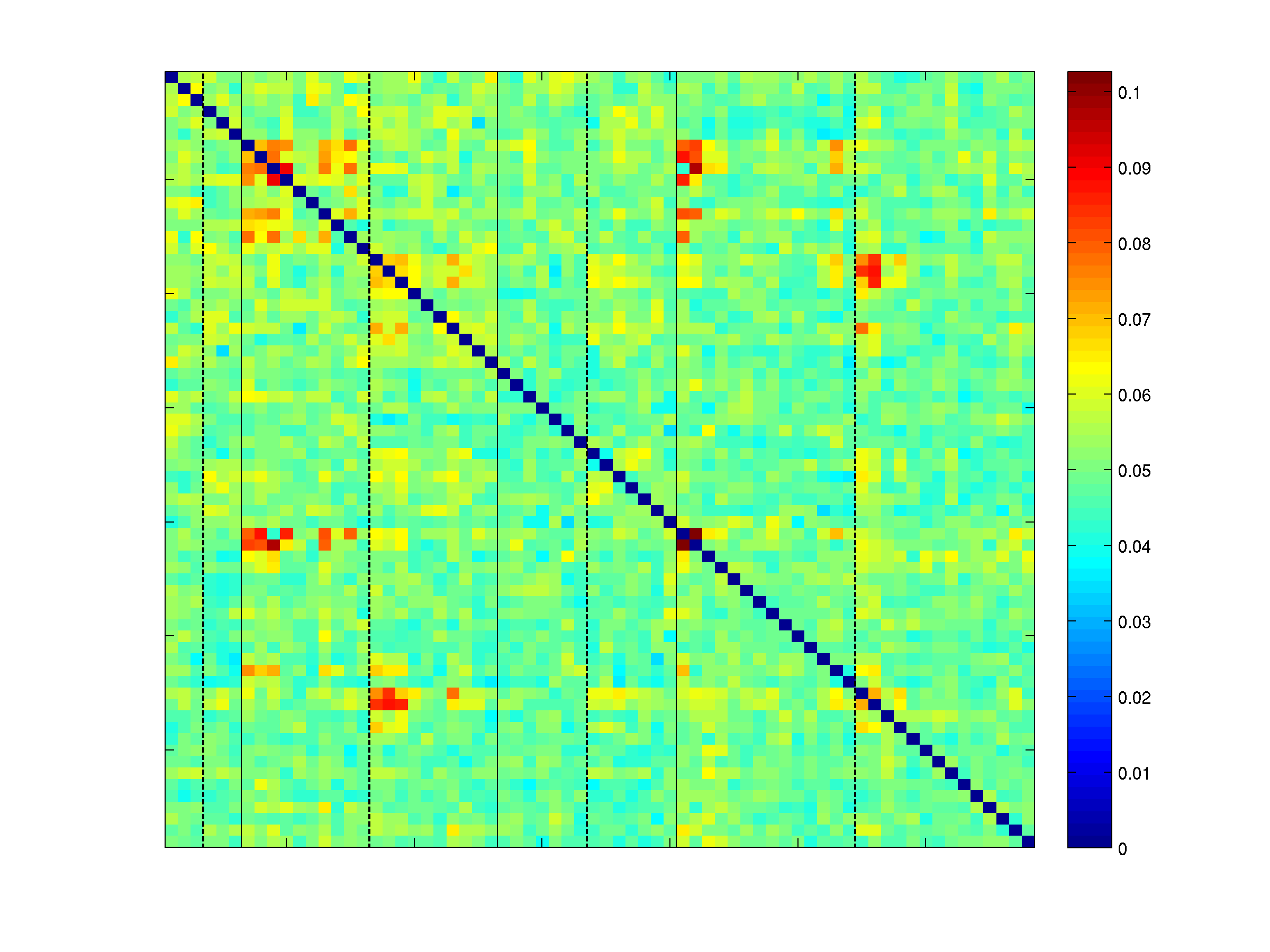*  **β** | *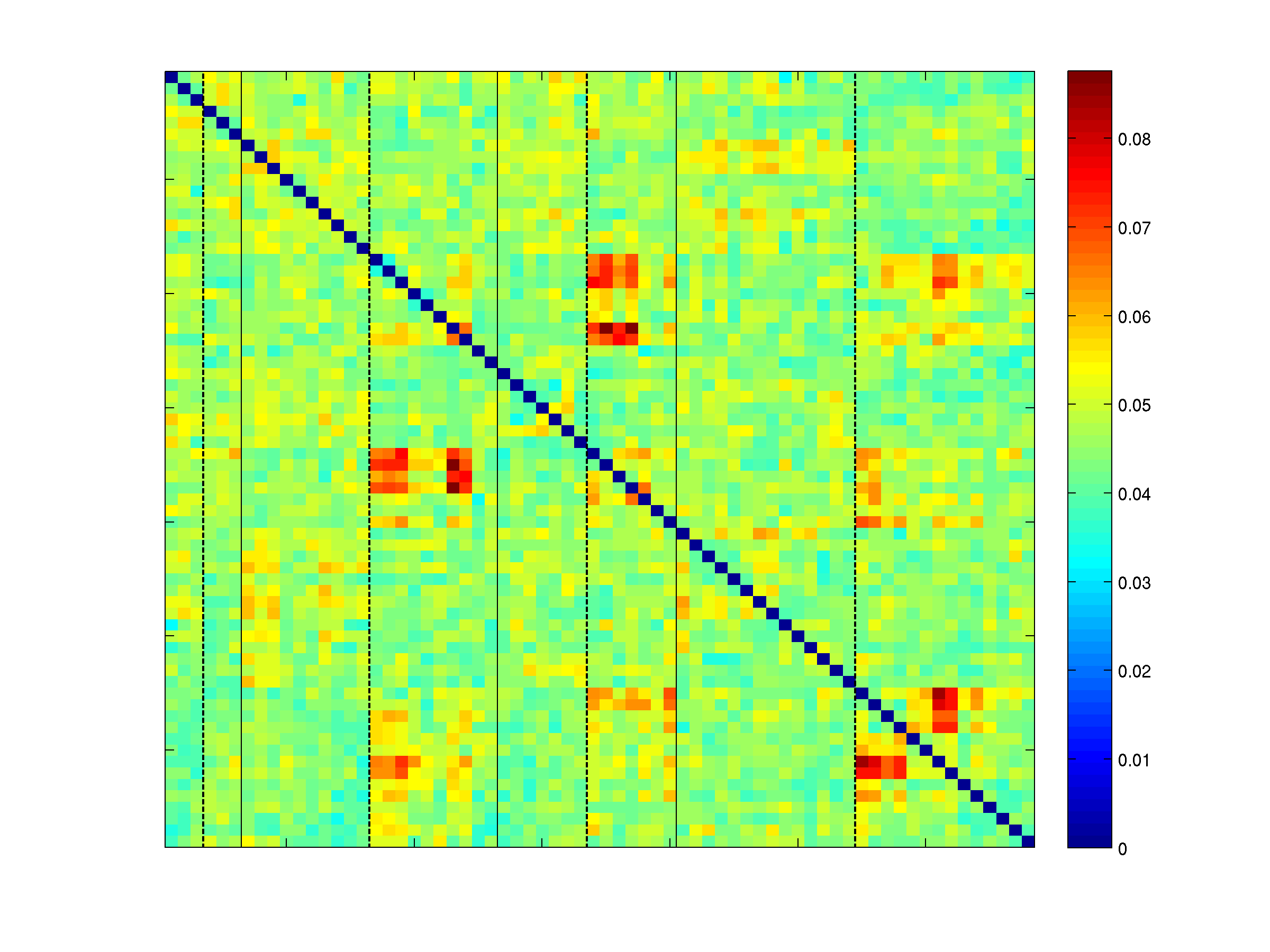*  **γ** |  |

**Supplementary Figure 1:** PLI-based adjacency matrices for delta, theta, alpha, beta and gamma bands. The separation between anatomical groupings (from left to right: occipital, parietal/central, temporal, frontal) is denoted by a solid line, the separation between left and right hemisphere within each anatomical grouping is denoted by a dotted line (see Appendix A for details).
