# Supplementary material for: Frequency-dependent functional connectivity within resting-state networks: An atlas-based MEG beamformer solution
Source: Neuroimage. 2012 Feb 15;59(4-2):3909–21. doi: 10.1016/j.neuroimage.2011.11.005 (PMC3382730; doi:10.1016/j.neuroimage.2011.11.005)
Supplement: Supplementary Fig. 2 — Mean PLI (left column) and mean relative power (right column) for delta (upper row) and theta bands (bottom row), displayed as a colour-coded map (unthresholded) on a schematic of the parcellated template brain. [file mmc2.doc]

*Functional connectivity and source power for the delta and theta bands*

Although the mean PLI values for the delta and theta bands did not reach statistical significance, some interesting observations were made from the unthresholded PLI maps and the power maps (Supplementary Figure 2).

In the delta band, the most strongly connected regions were found in the temporal and frontal lobe and the anterior cingulate. High power values were found in the same regions, with the exception that the power distribution was less prominent in the temporal lobe, and more prominent in pre-motor cortex, when compared to the connectivity pattern. The patterns of activation we observed are in agreement with previous reports of strong delta-band activation of the frontal lobe (Chen et al., 2008; Congedo et al., 2010) and anterior cingulate (Congedo et al., 2010).

The pattern of strongly connected, as well as that of strongly activated regions, in the theta band was more dispersed than for the other frequency bands. The most strongly connected regions were found in the temporal, and inferior frontal and parietal lobes (in agreement with Scheeringa et al. (2008) and in visual cortex. The most strongly activated regions were located in visual cortex, temporal, frontal and inferior parietal lobe, as well as in pre-motor and cingulate cortex. The strong frontal theta power is consistent with previous EEG findings (Chen et al., 2008; Srinivasan et al., 2006) (but not with their MEG findings (Srinivasan et al., 2006)). Similarly, the observed strong activations in temporal, frontal and inferior parietal lobe, as well as in pre-motor and cingulate cortex are all consistent with the results by Congedo and colleagues (Congedo et al., 2010). There are no previous reports of resting-state theta power in the occipital lobe, although our observations are in agreement with reports of event-related changes in theta power in a working-memory task (e.g. Raghavachari et al., 2001).

| Mean PLI | Mean Relative Power |
| --- | --- |
| **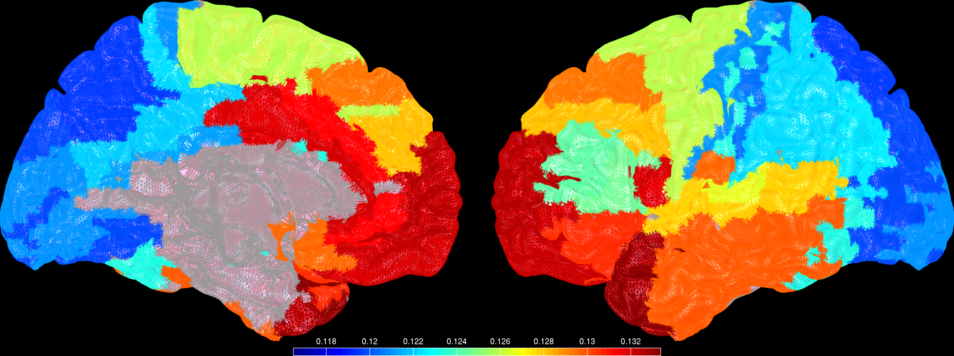**  **δ** | **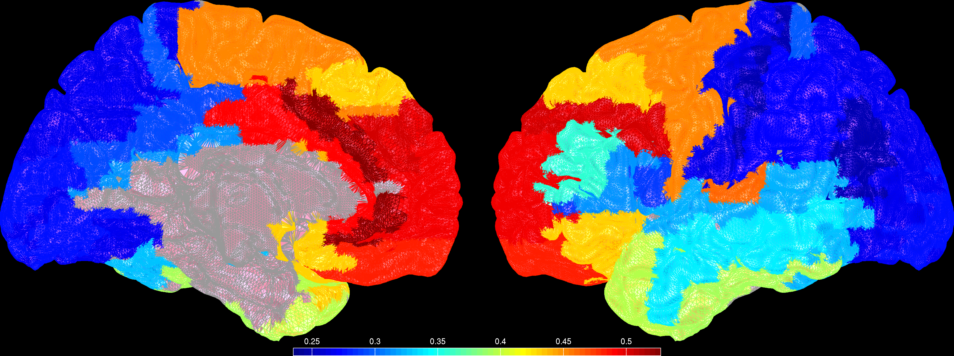** |
| **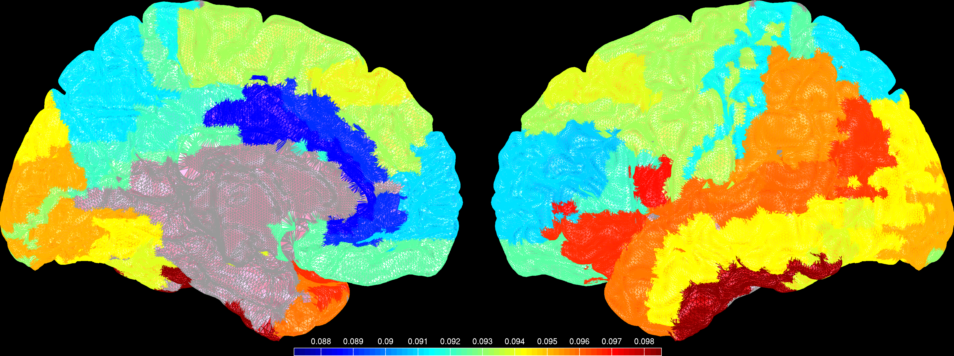**  **θ** | **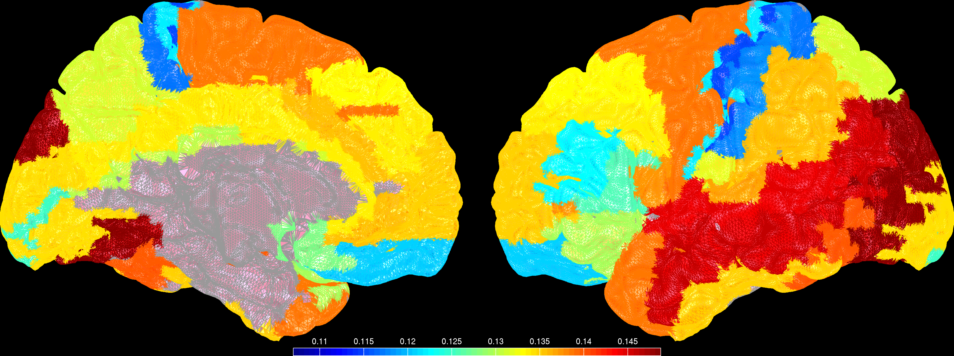** |

**Supplementary Figure 2:** Mean PLI (left column) and mean relative power (right column) for delta (upper row) and theta bands (bottom row), displayed as a colour-coded map (unthresholded) on a schematic of the parcellated template brain.
