# Supplementary material for: Frequency-dependent functional connectivity within resting-state networks: An atlas-based MEG beamformer solution
Source: Neuroimage. 2012 Feb 15;59(4-2):3909–21. doi: 10.1016/j.neuroimage.2011.11.005 (PMC3382730; doi:10.1016/j.neuroimage.2011.11.005)
Supplement: Supplementary Fig. 3 — Mean thresholded PLI (left column) and mean relative power (right column) for alpha1 (upper row) and alpha2 (bottom row), displayed as a colour-coded map on a schematic of the parcellated template brain. Note that alpha1 band PLI did not reach significance. [file mmc3.doc]

*Functional connectivity and source power for the alpha1 and alpha2 bands*

In order to be able to distinguish between the contribution from occipital alpha rhythms and central mu rhythms, we additionally analysed the alpha1 (8-10Hz) and alpha2 (10-13Hz) band separately. Significance of the PLI values (at the *p*<0.05 level) was again determined using phase-randomised surrogates. We found that there was no significant connectivity in the alpha1 band, and that the connectivity pattern for the alpha2 band (Supplementary Figure 3c) corresponded closely to the connectivity patterns found for the (broad) alpha band (Figure 4a). Comparing the power maps for the alpha1 and alpha2 bands (Supplementary Figures 3b, d) reveals that there is a shift in power from more occipital regions towards parietal/central areas when going from the alpha1 to the alpha2 band.

| Mean PLI | Mean Relative Power |
| --- | --- |
| **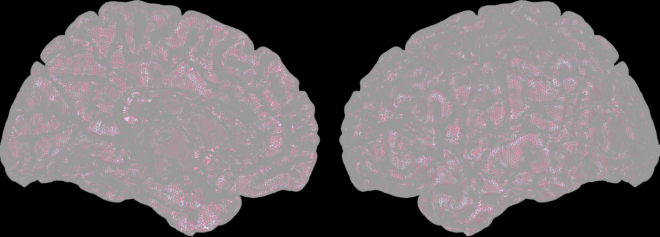**  **α1** | **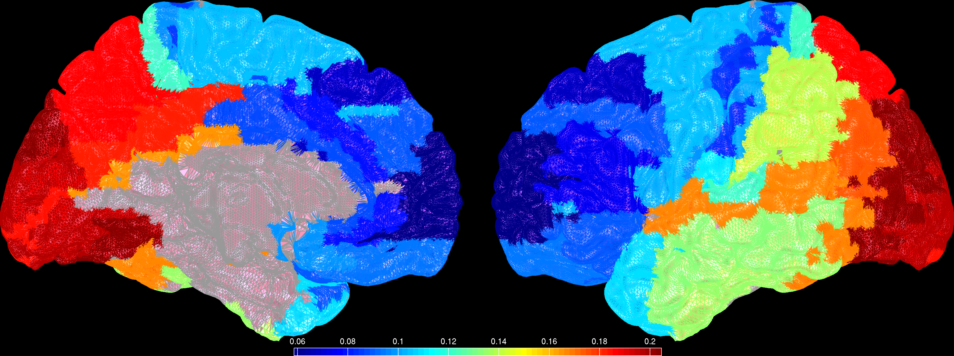** |
| **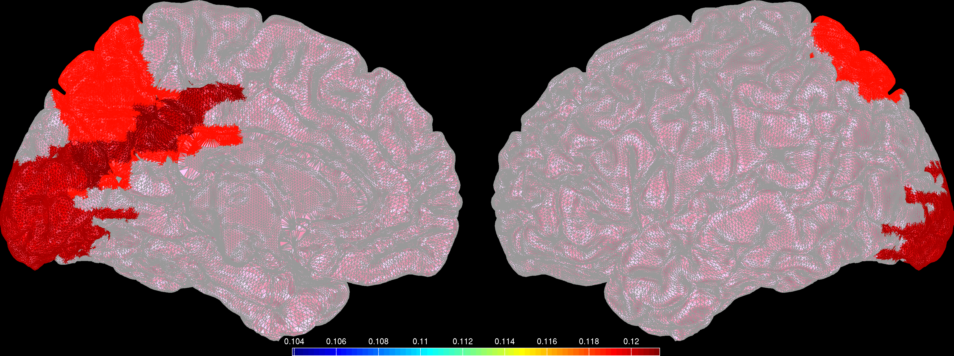**  **α2** | **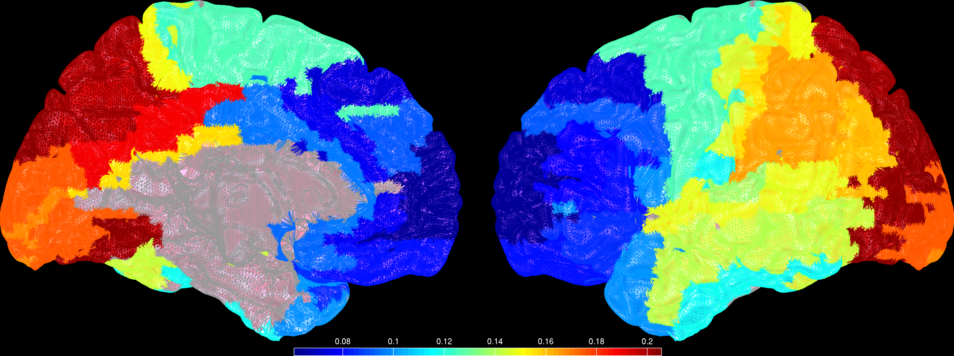** |

**Supplementary Figure 3:** Mean thresholded PLI (left column) and mean relative power (right column) for alpha1 (upper row) and alpha2 (bottom row), displayed as a colour-coded map on a schematic of the parcellated template brain. Note that alpha1 band PLI did not reach significance.
