# Supplementary material for: Frequency-dependent functional connectivity within resting-state networks: An atlas-based MEG beamformer solution
Source: Neuroimage. 2012 Feb 15;59(4-2):3909–21. doi: 10.1016/j.neuroimage.2011.11.005 (PMC3382730; doi:10.1016/j.neuroimage.2011.11.005)
Supplement: Supplementary Fig. 4 — Mean PLI (left column) and mean degree (using PLI values thresholded at 20% of maximum) (right column), for delta, theta, alpha, beta and gamma bands (top to bottom). [file mmc4.doc]

*Network characterisation*

The topology of a network can be characterised using graph theory, where the network is described as a set of nodes and edges (e.g. Bassett and Bullmore, 2009; Sporns, 2010; Stam and Reijneveld, 2007). Here, the nodes are the ROIs and the edges are the estimates of functional connectivity (the PC or PLI values). We constructed unweighted graphs, meaning that the edges were either present or absent. After thresholding the PLI values at 80% of the maximum value, we computed the degree of each node, which is simply the number of (remaining) edges connecting it to the rest of the network. The degree distribution, the probability distribution of degree over all nodes, provides important information about the structure of a network, for example whether network hubs exist (Barabasi and Albert, 1999).

Network analysis confirmed (Supplementary Figure 4) that, for all frequency bands, the patterns of average PLI corresponded closely to the patterns of node degree, indicating that large average PLI for a ROI is generally not due to a few very strong connections, but instead indicates that such a ROI is connected with many other ROIs.

| Mean PLI | Mean degree |
| --- | --- |
| **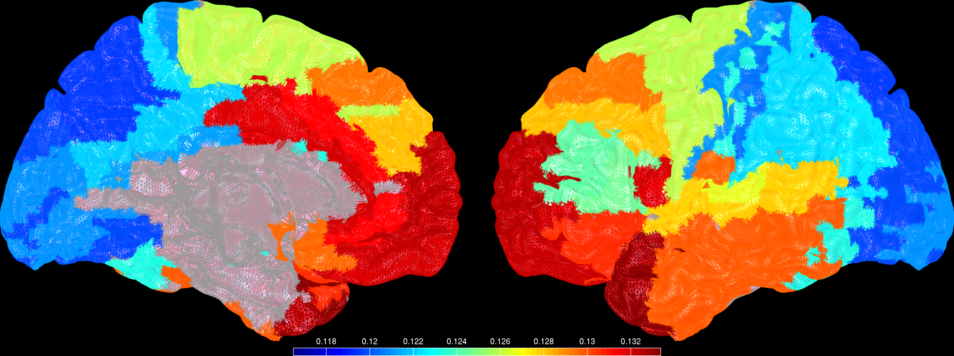**  **δ** | **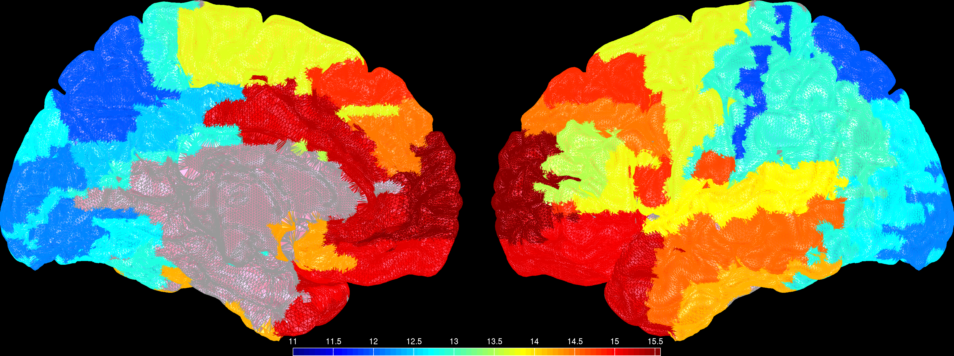** |
| **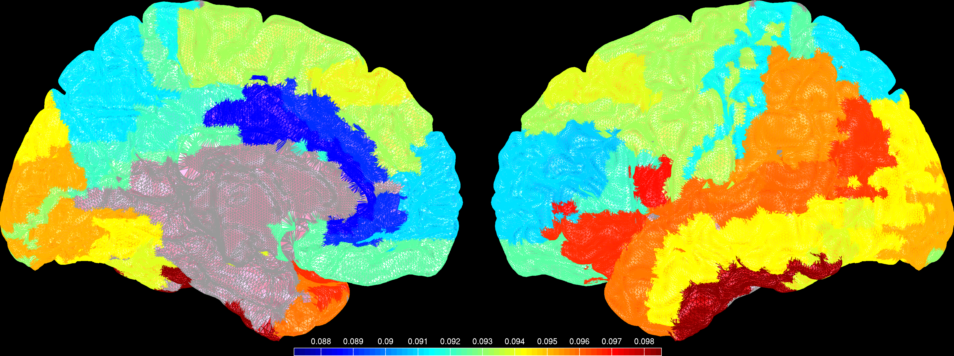**  **θ** | **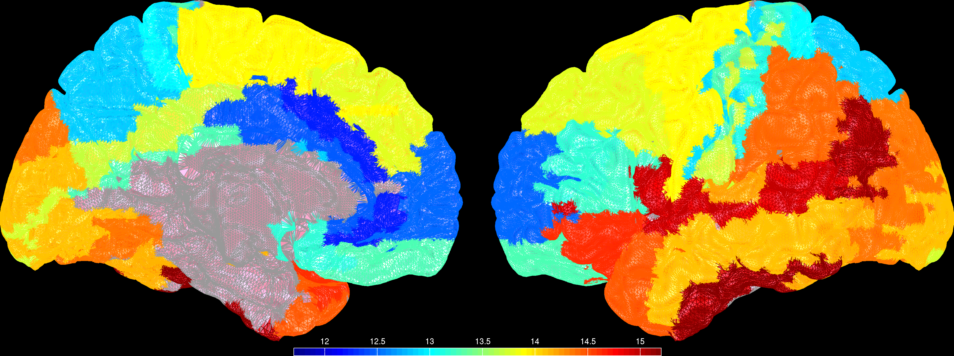** |
| **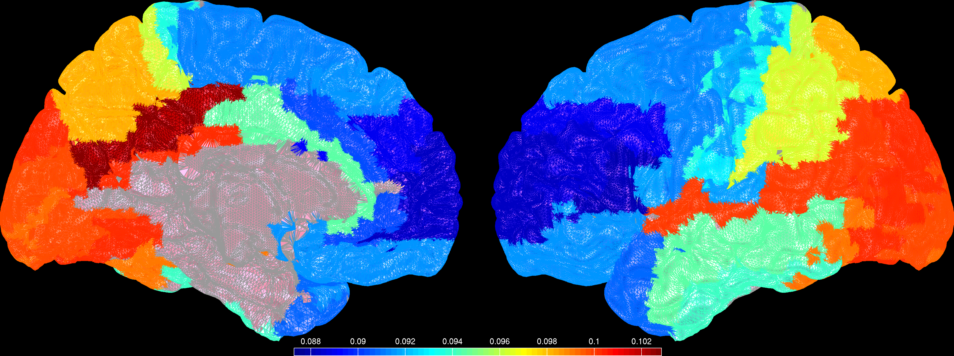**  **α** | **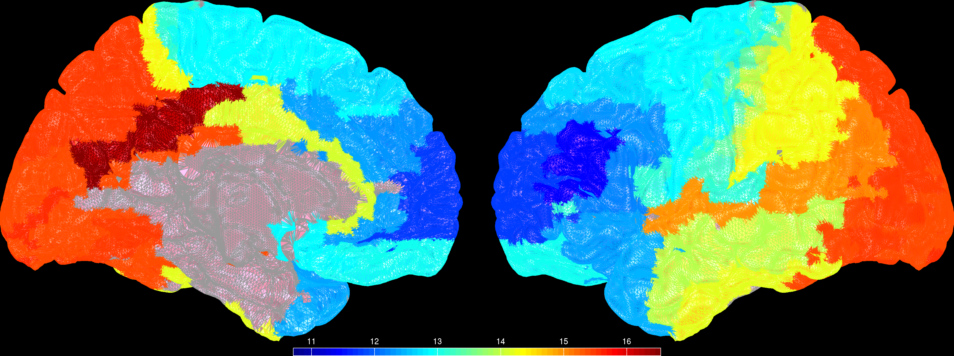** |
| **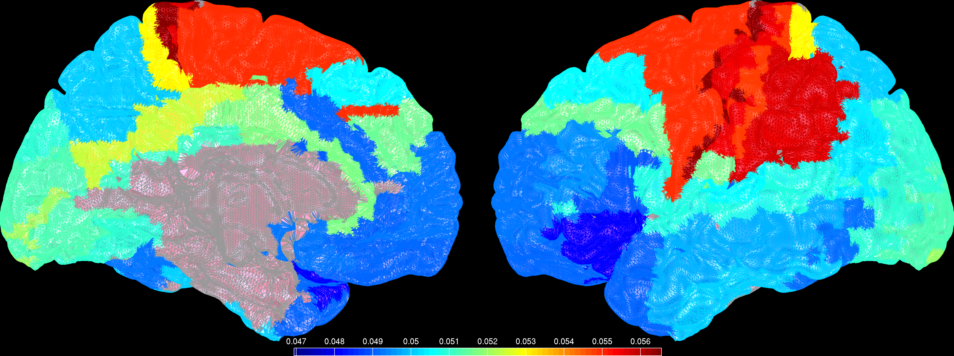**  **β** | **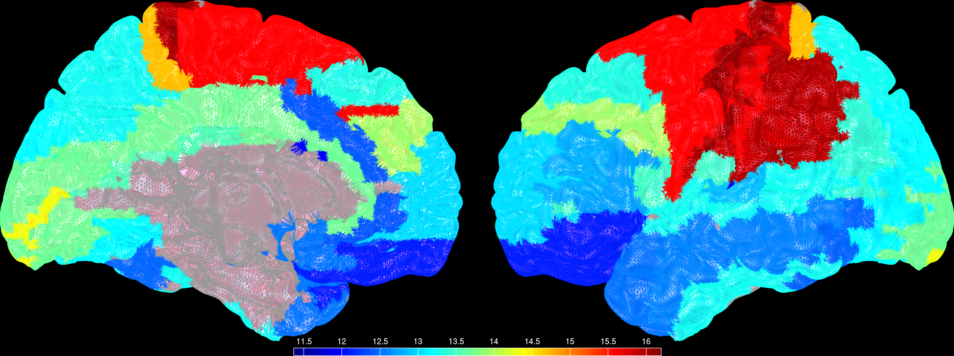** |
| **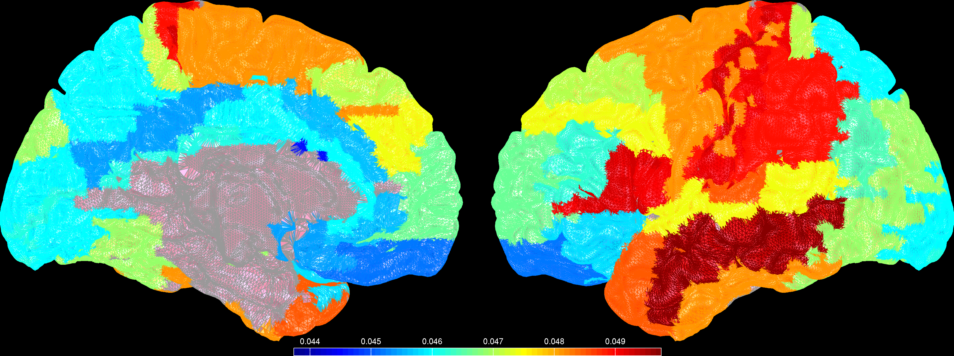**  **γ** | **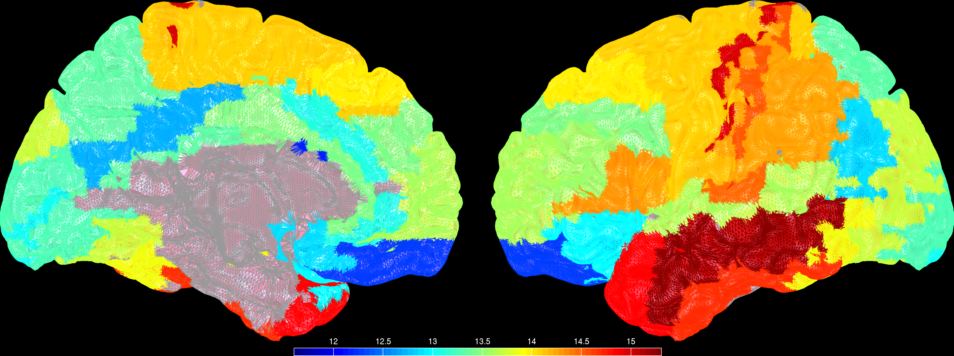** |

**Supplementary Figure 4:** Mean PLI (left column) and mean degree (using PLI values thresholded at 20% of maximum) (right column), for delta, theta, alpha, beta and gamma bands (top to bottom).
