# Supplementary material for: Frequency-dependent functional connectivity within resting-state networks: An atlas-based MEG beamformer solution
Source: Neuroimage. 2012 Feb 15;59(4-2):3909–21. doi: 10.1016/j.neuroimage.2011.11.005 (PMC3382730; doi:10.1016/j.neuroimage.2011.11.005)
Supplement: Supplementary Fig. 5 — a) Distance between peak voxels (in cm) averaged over subjects and frequency bands. The separation between anatomical groupings (from left to right: occipital, parietal/central, temporal, frontal) is denoted by a solid line, the separation between left and right hemisphere within each anatomical grouping is denoted by a dotted line (see Appendix A for details). The average distance was small between same ROIs (minimum distance was 0.7 cm for left BA3 and BA4), suggesting that the source reconstruction approach could not unambiguously determine whether the activity came from one or the other ROI (or both); b) scatter plot of the (squared) correlation between beamformer weights and the mean distance between peak voxels. Note that larger average weight correlation is found for peak voxels that are closer together, as is expected due to the correlation between lead fields for such voxels. [file mmc5.doc]

*Distance between ROIs*

For the selection of the voxel that represents a ROI, we chose, among all the voxels within a ROI, the voxel with maximum power within a frequency band. It is possible that for neighbouring ROIs, particularly when source activation spreads over multiple ROIs, the peak voxels for neighbouring ROIs are close together, i.e. share (almost) the same signal. We therefore computed the distance between the peak voxels, averaged over subjects and frequency bands (Supplementary Figure 5a), and found that, for some regions, this average distance was indeed small, suggesting that the source reconstruction approach could not unambiguously determine whether the activity came from one or the other ROI (or both). Importantly, PLI is insensitive to the spurious zero-lag interactions that could exist between voxels that are close together, hence the voxel selection will not lead to overestimates of (local) connectivity.

We further show (Supplementary Figure 5b) that there is a relationship between the beamformer weight correlation and spatial separation of the peak voxels, where larger average weight correlation is found for peak voxels that are closer together, as is expected due to the correlation between lead fields for such voxels.

| 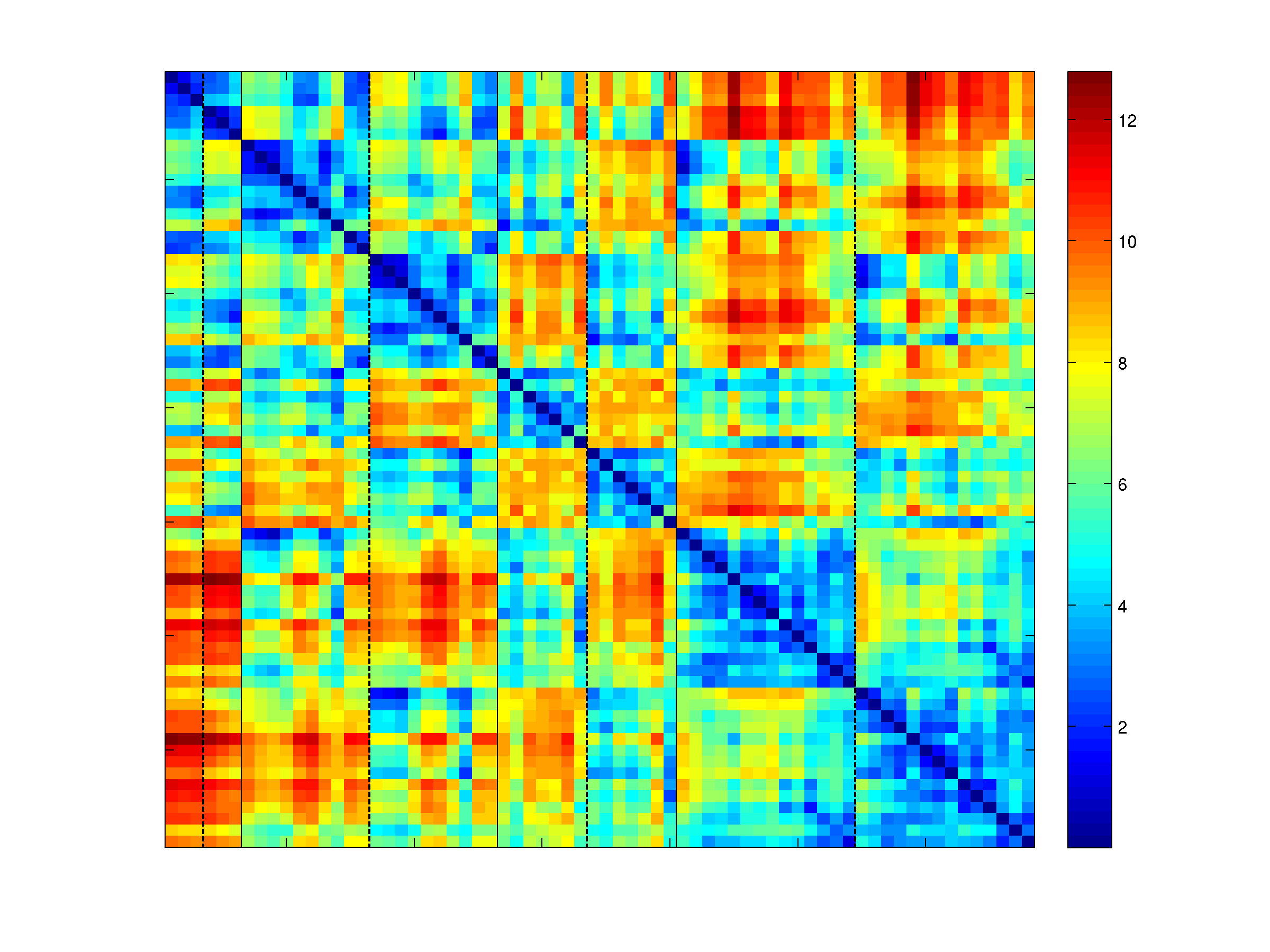  a | 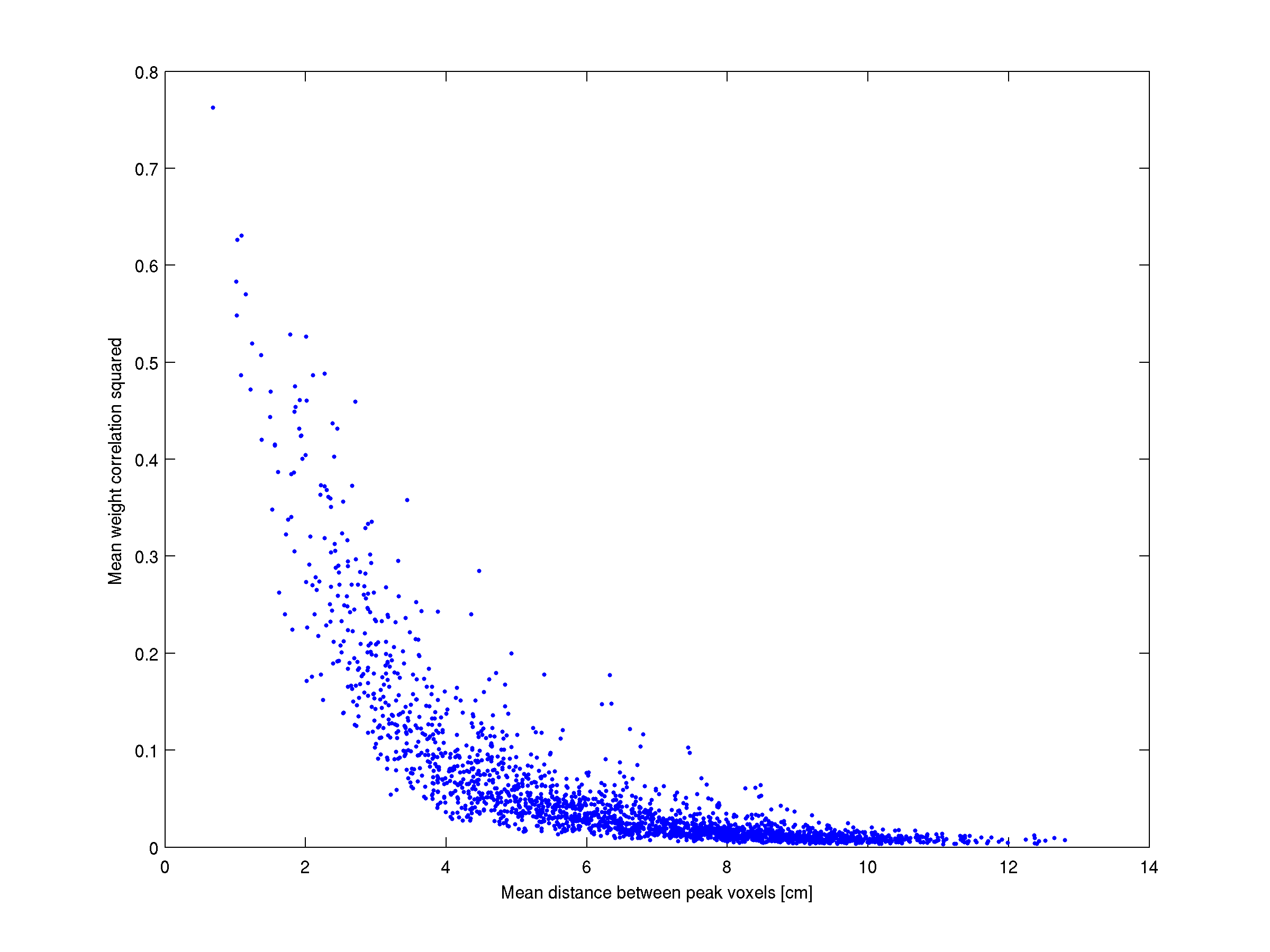  b |
| --- | --- |

**Supplementary Figure 5:** **a)** Distance between peak voxels (in cm) averaged over subjects and frequency bands. The separation between anatomical groupings (from left to right: occipital, parietal/central, temporal, frontal) is denoted by a solid line, the separation between left and right hemisphere within each anatomical grouping is denoted by a dotted line (see Appendix A for details). The average distance was small between same ROIs (minimum distance was 0.7 cm for left BA3 and BA4), suggesting that the source reconstruction approach could not unambiguously determine whether the activity came from one or the other ROI (or both); **b)** Scatter plot of the (squared) correlation between beamformer weights and the mean distance between peak voxels. Note that larger average weight correlation is found for peak voxels that are closer together, as is expected due to the correlation between lead fields for such voxels.
